# Recent progress in understanding protein and lipid factors affecting hepatic VLDL assembly and secretion

**DOI:** 10.1186/1743-7075-7-35

**Published:** 2010-04-27

**Authors:** Meenakshi Sundaram, Zemin Yao

**Affiliations:** 1 Department of Biochemistry, Microbiology and Immunology, Ottawa Institute of Systems Biology, University of Ottawa, 451 Smyth Road, Ottawa, Ontario K1H 8M5, Canada; 2 Department of Pathology and Laboratory Medicine, University of Ottawa, 451 Smyth Road, Ottawa, Ontario K1H 8M5, Canada

## Abstract

Excess lipid induced metabolic disorders are one of the major existing challenges for the society. Among many different causes of lipid disorders, overproduction and compromised catabolism of triacylglycerol-rich very low density lipoproteins (VLDL) have become increasingly prevalent leading to hyperlipidemia worldwide. This review provides the latest understanding in different aspects of VLDL assembly process, including structure-function relationships within apoB, mutations in *APOB *causing hypobetalipoproteinemia, significance of modulating microsomal triglyceride-transfer protein activity in VLDL assembly, alterations of VLDL assembly by different fatty acid species, and hepatic proteins involved in vesicular trafficking, and cytosolic lipid droplet metabolism that contribute to VLDL assembly. The role of lipoprotein receptors and exchangeable apolipoproteins that promote or diminish VLDL assembly and secretion is discussed. New understanding on dysregulated insulin signaling as a consequence of excessive triacylglycerol-rich VLDL in the plasma is also presented. It is hoped that a comprehensive view of protein and lipid factors that contribute to molecular and cellular events associated with VLDL assembly and secretion will assist in the identification of pharmaceutical targets to reduce disease complications related to hyperlipidemia.

## Introduction

Lipids of dietary origin as well as those stored in the adipose tissues act as energy sources for mammalian cells. Since lipids are hydrophobic in nature, mammals have evolved a mechanism such that the insoluble lipids are made soluble in the form of lipoproteins for transportation and delivery to various organs and tissues by the circulatory system. Formation and secretion of lipoprotein particles is primarily achieved in the liver (as VLDL) and in the intestine (as chylomicrons). The process involved in the assembly and secretion of hepatic VLDL or intestinal chylomicrons is complex and has been studied extensively for the past 2-3 decades. Lipid and protein factors that affect various steps during the assembly and secretion of VLDL and chylomicrons have been identified. The assembly process of hepatic VLDL is initiated in the endoplasmic reticulum (ER) as soon as apoB-100 is translated and translocated into the lumenal side where the elongating apoB-100 polypeptide chain recruits various lipids co-translationally. Each VLDL is composed of one molecule of apoB-100, multiple copies of other apolipoproteins, together with varied amounts of triacylglycerol (TAG) and cholesteryl esters, depending upon the size of resulting particles. Cellular and molecular mechanisms by which different lipid and protein components are brought together for VLDL assembly are not fully understood and remain to be defined. A protein factor other than apoB that is absolutely required for VLDL assembly is the microsomal triglyceride-transfer protein (MTP). The obligatory role of MTP in VLDL assembly/secretion is exemplified by human familial abetalipoproteinemia, characterized by nearly a complete absence of apoB-containing lipoproteins including VLDL (and chylomicrons as well). Available evidence indicates that among different lipid and protein constituents of VLDL, the availability of functional apoB-100 and TAG are by far the most critical for the assembly of secretion-competent VLDL within the ER lumen. An array of protein factors involved in secretory protein translation and translocation across the ER membrane are responsible for initial apoB-100 folding to attain lipid-binding capability within the microsomal lumen. Pathological conditions that disfavor apoB-100 folding or binding of lipids to apoB will result in aborted VLDL assembly and premature intracellular degradation of apoB-100 during or after translation.

## Structural and functional elements within apoB-100

**Figure 1:**
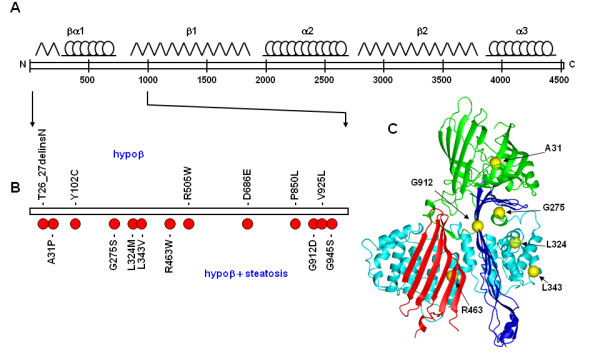
**Model of the N-terminal of apoB and positions of FHBL mutations**. A, schematic diagram of apoB-100, with predicted locations of βα1, β1, α2, β2, and α3 domains are shown on the *top*. B, positions of non-truncating FHBL mutations within the N-terminal 1,000 amino acids of apoB-100. Mutations associated with phenotype of hypobetalipoproteinemia (*hypoβ*) are shown on the *top*, whereas mutations associated with phenotype of both hypoβ and hepatic steatosis are shown *below*. C, proposed homologous model of the N-terminal ~930 amino acids of apoB. The β-barrel, α-helical, and β sheet (C-sheet and A-sheet) are highlighted in *green*, *cyan*, *red*, and *blue*, respectively. Locations of Ala^31^, Gly^275^, Leu^324^, Leu^343^, Arg^463^, and Gly^912 ^within the modeled βα1 domain are shown as *yellow spheres*.

The human *APOB *gene, located on the distal end of the short arm of chromosome 2 (2p23-2p24), encodes a ~20 kb mRNA that is translated into the full-length apoB-100 consisting of 4,536 amino acids in the liver [1-3]. A truncated form of apoB, known as apoB-48, represents the N-terminal 48% of apoB-100 and is produced in the intestine by an mRNA editing mechanism [4]. In humans, apoB-100 and apoB-48 are obligatory proteins for the assembly of respective hepatic VLDL and intestinal chylomicrons [5]. In mouse and rat, the liver synthesizes apoB-48 in addition to apoB-100 [6]. Because of their enormous size, extreme hydrophobicity along with varied extents in lipid-binding, the 3-D structure of apoB-100 or apoB-48 has not been solved at the atomic level. However, attempts have been made, using various algorithms, to compute the structures of various domains of apoB-100. The modeled human apoB-100 molecule is composed of five domains enriched with alternating amphipathic α-helices and amphipathic β-strands, designated βα1-β1-α2-β2-α3 [7]. Various domains and their approximate locations in apoB-100 are depicted in Fig. 1A. Moreover, based on the homology to lamprey lipovitellin, a modeled structure for the N-terminal ~930 amino acids of human apoB-100 has been proposed [8,9]. This model predicts a βα1 domain structure consisting of β-barrel (the first 264 residues) and α-helical bundle (residues 292-593), followed by two amphipathic β-sheets termed C sheet (residues 611-782) and A sheet (residues 783-930), respectively, that may form a lipid-binding pocket [10]. Scanning transmission electron microscopy studies have provided direct evidence that nanogold-labeled apoB fragment (apoB6.4-17) interacted with lipids [11]. A model of human apoB-100 associated with low density lipoprotein (LDL) has been obtained using images captured by electron cryomicroscopy, in which a single apoB-100 molecule with its α-helix and β-sheet rich domains across the LDL surface is proposed [12].

The N-terminal region of apoB contains several binding sites for MTP, an apoB-specific molecular chaperone essential for apoB-100 to fold properly during VLDL assembly/secretion [13,14]. Multiple MTP-binding sites have been identified within the N-terminal βα1 domain of apoB [13,15,16], and the affinity of MTP-binding is inversely related to the apoB polypeptide length [17]. Apart from binding to MTP, the βα1 domain of apoB-100 also binds to scavenger receptors in human macrophages [18] and to lipoprotein lipase [19]. The β1 domain (amino acids 827-1880) of apoB-100 is predominantly made of amphipathic β strands and is involved in irreversible lipid-binding [20,21]. The β2 domain located at the C-terminal half of apoB-100 possesses LDL receptor-binding property [22,23]. The role of the intervening amphipathic α-helix enriched domain α2 and the C-terminal α3 of apoB-100 may represent a flexible region that imparts elasticity to the molecule allowing recruitment of various amounts of core lipids [24].

The amino acid sequences within apoB-100 essential for lipoprotein assembly and secretion have been investigated extensively using two approaches, namely truncation mutagenesis and chimeric protein expression analysis. Incremental truncation of human apoB polypeptide from the carboxyl terminus successively decreased its ability to form buoyant lipoprotein particles [25,26]. Under lipid rich conditions, the ability of apoB to assemble VLDL resides at the length transition between the N-terminal ~30% and ~40% of the polypeptide, a region enriched with amphipathic β strands (*i.e*. the β1 domain) [26]. Studies with chimera proteins, in which segments of apoB derived from the β1 domain were fused with apoA-I, have shown that inclusion of the amphipathic β strands conferred the ability to assemble VLDL [26]. The amino acid sequences within the β1 domain also influence the rate of apoB translocation across the ER membrane and its susceptibility to proteasomal degradation [27] (for apoB degradation, see below). These studies suggest that the amino acid sequences within the β1 domain are important for lipid-binding in the VLDL assembly process.

Human apoB-100 undergoes several posttranslational modifications, including disulfide bond formation, N-linked glycosylation, and palmitoylation. Mutational analysis has shown that among six disulfide linkages within the βα1 domain, the two involving Cys^51^/Cys^70 ^and Cys^218^/Cys^234 ^are essential for apoB-lipoprotein assembly and secretion [28,29]. Results from the cysteine mutagenesis studies corroborate with data obtained from dithiothreitol treatment experiment in which abolishing apoB disulfide bond formation with the reducing agent resulted in decreased apoB secretion [30].

There are 20 potential N-linked glycosylation sites within human apoB-100, of which 16 Asn residues are conjugated with oligosaccharides in apoB-100 associated with plasma LDL [31]. The requirement of Asn^158^, Asn^956^, Asn^1341^, Asn^1350^, and Asn^1496 ^residues within the amino terminus of apoB-100 has been determined by mutagenesis experiments; results from these studies suggested that the loss of N-glycans, particularly at Asn^1496^, resulted in decreased stability of apoB and reduced secretion of TAG-rich lipoproteins [32]. Thus, in addition to lipid binding sequences of apoB, the N-linked oligosaccharides conjugated to apoB also play a role conferring the posttranslational stability and the ability to assemble lipoproteins.

Palmitoylation of human apoB is observed in plasma LDL [33] as well as apoB found in cultured hepatic cell lines [34,35]. The 16-carbon fatty acid palmitate is covalently linked to cysteine residues via thioester bonds. Mutagenesis studies of the four cysteine residues Cys^1085^, Cys^1396^, Cys^1478^, and Cys^1635 ^suggest that the lack of palmitoylation within the N-terminal region of apoB does not compromise the ability of apoB-48 to assemble lipoproteins [36]. However, decreased secretion of the short truncation mutant apoB-29 was observed as a result of lack of palmitoylation [35]. It is likely that an interplay exists between the length of amphipathic lipid-binding sequences and palmitoylation of apoB that regulate TAG-rich lipoprotein assembly and secretion.

Many mutations in the *APOB *gene have been characterized and found to affect the plasma concentrations of apoB, TAG and cholesterol. The most characterized *APOB *mutations are the ones found in familial hypobetalipoproteinemia (FHBL) [37]. FHBL is inherited in a Mendelian fashion as an autosomal dominant trait. Heterozygote FHBL individuals invariably have plasma cholesterol, TAG, LDL-cholesterol, and apoB at levels approximately 1/3 of normal, whereas homozygote FHBL subjects have barely detectable levels of apoB. Individuals with FHBL have reduced risk of cardiovascular diseases presumably owing to low plasma apoB and cholesterol concentrations [38]. Various truncated forms of apoB, such as apoB-32 [39], apoB-55 [40], apoB-61 [41], apoB-75 [42], and apoB-83 [43] to name a few, are found in human subjects displaying FHBL phenotype as a result of nonsense mutations occurring throughout the apoB polypeptide. To date, novel truncating mutations within *APOB *are continually being identified in FBHL patients devoid of cardiovascular diseases [44]. Cell culture and transgenic mouse studies with a variety of truncated apoB forms (ranging from apoB-15 to apoB-94) showed that most of the C-terminally truncated apoBs were secreted as efficiently as normal apoB-100 or apoB-48 [25,26,45,46].

Non-truncating mutations in *APOB *also cause FHBL; most of these nonsynoymous mutations occur within the βα1 domain of apoB [47-49]. Biochemical analysis has shown that unlike the apoB truncation mutants whose secretion efficiency was normal, the nonsynoymous apoB mutants exhibited markedly decreased secretion from transfected cells and abnormal retention of the mutant proteins within the ER or Golgi [49,50]. Twelve non-truncating mutations within the N-terminal 1,000 amino acids of apoB-100, some of them associated with hypobetalipoproteinemia and some confounded with hepatic steatosis, are shown in Fig. 1B. Table 1 summarizes the seven non-truncating apoB mutations that have been biochemically characterized. For example, the mutant A31P apoB proteins identified in an Italian FHBL subject showed severely impaired secretion and augmented intracellular degradation by proteasomes and autophagy [50]. The identification of missense mutations within the βα1 domain of apoB indicates the structural and functional importance of this domain, and also provides additional explanation for the early observations that recombinant apoB segments lacking the N-terminal 1,000 amino acids were either secreted poorly or not secreted at all [25,26]. Position of the amino acids corresponding to FHBL mutations within the predicted βα1 domain of apoB is depicted in Fig. 1C.

**Table 1:** Biochemically characterized *APOB *missense mutations in FHBL

Mutation	Predicted location	Hepatosteatosis	Secretion	Retention	Reference
A31P	β-barrel	yes	<5% normal	Golgi	[50]
G275S	α-helix	yes	60% normal	n.d.	[50]
L324M	α-helix	yes	50% normal	n.d.	[50]
L343V	α-helix	yes	50% normal	ER	[48]
R463W	α-helix	yes	50% normal	ER	[47]
G912D	β-sheet	yes	nearly normal	n.d.	[50]
G945S	β-sheet	yes	50% normal	n.d.	[50]

n.d, not determined

## MTP and VLDL assembly

MTP is a heterodimer consisting of a 97-kDa lipid-binding and -transfer subunit [51] and a 55-kDa protein disulfide isomerase (PDI) [52] that is not required for lipid-transfer activity [53]. MTP is predominantly expressed in the hepatocytes and enterocytes where it transfers neutral lipids required for the assembly of apoB containing lipoproteins such as VLDL in the liver and chylomicrons in the intestine. Mutations leading to loss of MTP activity is linked to familial abetalipoproteinemia [54] in which the affected individuals have undetectable levels of apoB in the plasma. Structural studies of various domains within MTP have revealed a lipid-binding cavity resembling those found in intracellular lipid-binding proteins. The entrance of the cavity contains two conserved helices (helix-A: amino acids 725-736 and helix-B: amino acids 781-786). Mutational analysis of the helices has shown that helix A is required for the acquisition of lipids from phospholipid membranes whereas helix B plays a role in transferring lipids to the lipid-binding cavity. Mutations introduced in either of the helices resulted in abolition of lipid binding, which provides an explanation for abetalipoproteinemia found in humans carrying point mutations in the *MTTP *gene [55].

Availability of lipids in the vicinity of MTP is crucial for proper acquisition and transfer of lipids to the site of VLDL assembly within the ER. Lack of sufficient lipids supply compromises VLDL assembly and maturation, resulting in poorly lipidated apoB polypeptides that are prone to co- or post-translational degradation [56]. Inhibition of MTP with chemical inhibitor results in a similar outcome leading to the failure of VLDL assembly [57]. Studies with rat hepatoma McA-RH7777 cells have suggested that the early stage of lipid assembly initiated during apoB translation and translocation is facilitated by the MTP activity. However, during the later stage of VLDL assembly where bulk TAG is incorporated, the activity of MTP appears not essential [58]. This stage-dependent feature of MTP activity during VLDL assembly has also been observed in human hepatoblastoma HepG2 cells [59] and for the assembly of VLDL by McA-RH7777 cells transfected with recombinant human apoB-48 [60]. Graded inactivation of MTP with increasing doses of MTP inhibitor has revealed that the assembly and secretion of TAG-rich VLDL_1 _(*S*_*f *_> 100) and VLDL_2 _(*S*_*f *_20-100) are more dependent on MTP activity than TAG-poor particles such as intermediate density lipoproteins (IDL) and LDL [57]. Moreover, the requirement of MTP activity is more pronounced for apoB-100 than for apoB-48, thus MTP inhibition effectively decreases apoB-100 and TAG secretion but has little effect on secretion of lipid-poor apoB-48 [61]. Lipid pulse-chase analysis carried out in rat primary hepatocytes after MTP activity was inhibited revealed delayed removal of TAG from the membranes of ER and Golgi. Inactivation of MTP did not affect lipolysis or subsequent re-esterification of TAG but the re-esterified TAG was not secreted as VLDL [62]. These cell culture studies suggest that MTP facilitates recruitment of TAG from the ER/Golgi membranes during VLDL assembly without affecting TAG biosynthesis.

In addition to cell culture studies, the role of MTP in VLDL assembly and secretion has also been investigated using genetically modified mice. The liver specific *Mttp *knockout mice displayed a phenotype equivalent to the human abetalipoproteinemia (*i.e. *absence of plasma apoB-100 and low levels of apoB-48) and were resistant to hypercholesterolemia induced by high cholesterol diet [63]. Ultrastructural analysis of liver specimen of the *Mttp *knockout mice has revealed absence of lipid particles within the ER/Golgi lumen, whereas in wildtype control mice the lipidic bodies of VLDL size were readily observable [64]. These results raise the possibility that the MTP activity is required for mobilization and partitioning of TAG substrates into the microsomal lumen for VLDL assembly.

Regulation of MTP expression is achieved by multiple factors that are closely related to fatty acid metabolism and changes in MTP expression is invariably associated with altered hepatic VLDL production. Studies with L35 cells, derived as a single cell clone from the rat hepatoma FAO cells, have shown that the inability of these cells to assemble or secrete VLDL resulted from co-repression of MTP and L-FABP (liver fatty acid binding protein) by chicken ovalbumin upstream promoter transcription factor II that occupied the DR1 promoter region of *L-FABP *and *Mttp*. Under these conditions, although VLDL secretion was blocked, the liver did not develop steatosis. In parental FAO cells, the DR1 promoter region of *L-FABP *and *Mttp *was occupied by peroxisome proliferator-activated receptor α (PPARα)-retinoid × receptor α (RXRα), and the transcription of L-FABP and MTP were normal, hence the assembly and secretion of VLDL was ensured [65]. In the leptin-deficient obese *ob*/*ob *mice, hepatosteatosis and insulin resistance (partly as a result of excessive fatty acid influx into the liver) were ameliorated by deletion of an orphan receptor small heterodimer partner (SHP) [66]. This study has revealed that SHP acts as a repressor of MTP mRNA transcription by liver receptor homolog-1 and therefore decreases MTP activity and VLDL secretion in *ob*/*ob *mice [66]. The MTP expression is also regulated by FoxO1, a transcription factor that plays a role in muscle cell growth and differentiation [67]. Studies with HepG2 cells have suggested that FoxO1 could bind to *Mttp *promoter region and directly stimulate MTP expression. It was also noticed that insulin treatment suppressed FoxO1 mediated MTP expression, and the insulin effect could be abolished by deleting or mutating the FoxO1 binding site within the *Mttp *promoter [68]. In addition, MTP expression can also be activated by Foxa2 together with PPARγ coactivator β (PGC-1β), resulting in increased VLDL secretion [69].

In summary, cell culture and transgenic mouse studies indicate that alteration in MTP activity has a direct effect on VLDL production. Attenuating MTP expression and/or activity without eliciting hepatosteatosis has been a long sought-after strategy in treating dyslipidemia related to overproduction of VLDL.

## Fatty acid substrates affecting VLDL assembly and secretion

TAG utilized for VLDL assembly and secretion originates from multiple biosynthesis pathways. Fatty acyl chains used for TAG synthesis can be derived from *de novo *synthesis or from hydrolysis of existing TAG, presumably catalyzed by triacylglycerol hydrolase in the liver [70]. These fatty acyl chains are also used for phospholipid biosynthesis. In addition, hydrolysis of existing phospholipids associated with intracellular membranes [71,72] or exogenous phospholipids associated with high density lipoproteins (HDL) [73], also provides fatty acyl chains for TAG synthesis and secretion.

Comparative analyses have shown that the chemical nature of fatty acids, such as the acyl chain length and the extent of unsaturation (*i.e. *the number and position of double bonds) of acyl chains, have a profound impact on VLDL assembly and secretion. For instance, studies with chicken primary hepatocytes treated with different medium-chain fatty acids have shown that octanoate (8:0), decanoate (10:0), or dodecanoate (12:0) significantly decreased VLDL-apoB secretion compared to palmitate (16:0) [74]. The inhibitory effect of octanoate (usually present in coconut and breast milk) on VLDL was linked to suppression of apoB gene expression and apoB100 protein synthesis without affecting intracellular degradation of apoB [75].

Contrasting saturated fatty acids with mono- or poly-unsaturated fatty acids has shown that certain fatty acid species are preferentially utilized over others for VLDL assembly and secretion. McA-RH7777 cells, when cultured in the presence of exogenous oleate (18:1 n-9), secreted TAG-rich VLDL many fold higher as compared to no oleate supplementation [60,70,76,77]. However, when the cells were treated with poly-unsaturated fatty acids such as eicosapentaenoic acid (20:5 n-3), assembly and secretion of TAG-rich VLDL was significantly decreased [72]. The impaired secretion of apoB in cells treated with docosahexaenoic acid (22:6 n-3) was associated with increased oxidation, aggregation and autophagic degradation of apoB [78]. In rat primary hepatocytes, treatment with chylomicron remnants rich in saturated fatty acids or n-6 polyunsaturated fatty acids were more potent in promoting TAG-rich VLDL secretion than those rich in monounsaturated fatty acids [79]. HepG2 cells treated with a mixture of conjugated linoleic acids synthesized and secreted significantly less apoB-100 as compared to treatment with saturated or polyunsaturated fatty acids [80]. These studies suggest strongly that the characteristics of different fatty acids have distinct impacts on hepatic VLDL secretion.

The differential effects of fatty acid feeding on VLDL production has also been observed *in vivo *in animal studies. For example, transgenic mice lacking LDL receptor (*Ldlr*^-/-^) and expressing only apoB-100 responded differently in VLDL production to diet supplemented with echium oil (rich in 18:4 n-3), fish oil (rich in 20:5 n-3 and 22:6 n-3) or palm oil (enriched in 16:0) [81]. Feeding mice with diet supplemented with or without essential fatty acids also resulted in production of VLDL particles with different size and different catabolic rates [82].

Mechanisms by which certain fatty acid species preferentially promote VLDL secretion remain to be defined. Different fatty acid species may affect transcription of genes involved in TAG synthesis [81], which in turn may affect VLDL assembly and secretion. Acutely, treatment with different fatty acids may generate TAG molecules that are differentially utilized for VLDL assembly and secretion. For examples, studies with McA-RH7777 cells have shown that TAG derived from oleic acid (18:1 n-9) was partitioned into microsomes and was effectively secreted as VLDL, whereas TAG derived from eicosapentaenoic acid (20:5 n-3) was stored in the cytosol and was poorly secreted [72]. The discriminative usage of certain TAG molecules for VLDL assembly and secretion may be attributable to compartmentalization [72]. However as discussed below, because fatty acyl chains are also substrates for membrane phospholipid biogenesis, it is possible that a membrane milieu that supports assembly, trafficking, and maturation of TAG-rich VLDL is imparted by certain fatty acid species.

Besides the chemical nature of fatty acid species, the duration and concentration of fatty acid treatment also influence VLDL assembly and secretion. This could be as a result of either fatty acid-induced lipotoxicity or fatty acid-mediated transcription activation of lipid metabolism genes. Feeding *Ldlr*^-/- ^mice with conjugated linoleic acid (10*trans*,12*cis*-18:2) for a short period of time resulted in hypertriglyceridemia with elevated plasma VLDL and HDL. However, long term feeding with the same fatty acid decreased plasma VLDL associated TAG, probably attributable to upregulated expression of hepatic VLDL receptor, lipoprotein lipase, and fatty acid translocase that could assist in the clearance of lipoproteins from circulation [83]. Prolonged incubation of McA-RH7777 cells with high concentrations of oleic acid also resulted in decreased apoB-100 secretion and increased apo-B100 degradation through proteasomal and non-proteasomal pathways, as a result of ER stress associated with massive accumulation of intracellular TAG [84]. Thus, overloading of lipids to the extent that exceeds the capacity of hepatocytes to effectively assemble and secrete VLDL would lead to impaired TAG secretion and cause hepatosteatosis. In addition to fatty acid-associated lipotoxicity, it has been reported recently that hepatic overexpression of apoB also induce hepatic ER stress and insulin resistance [85]. The molecular mechanisms by which excess fatty acid influx and overproduction of apoB-100 lead to ER stress and altered insulin signal transduction merits further investigation.

## Glycerolipid biogenesis and VLDL assembly and secretion

Identification and characterization of glycerolipid synthesis gene products have accelerated our understanding on the impact of hepatic lipid synthesis and/or their availability on VLDL assembly and secretion. The rate limiting step in the *de novo *biosynthesis of TAG and phospholipids is catalyzed by phosphatidate phosphatase-1 (PAP-1) [86], which converts phosphatidate to diacylglycerol that is subsequently utilized for the synthesis of TAG and phospholipids such as phosphatidylcholine (PC) and phosphatidylethanolamine (PE). In mammals, PAP-1 is encoded by the lipin gene family consisting of lipin-1, -2 and -3 [87,88]. In humans, lipin-1 is highly expressed in skeletal muscles and adipose tissues; lipin-2 and -3 are expressed in brain, liver, jejunum, placenta, and adipose tissues [88]. The *Lpin1 *gene encodes two alternatively spliced isoforms lipin-1α and -1β [89], and the expression of *Lpin1 *is upregulated by glucocorticoids and suppressed by insulin [90]. Transient expression of lipin-1α or -1β in McA-RH7777 cells resulted in increased synthesis and secretion glycerolipids under basal or lipid-rich conditions [91]. Secretion of TAG as VLDL_1 _was increased upon lipin-1α or -1β expression when cells were cultured under lipid-rich conditions [91]. These results were not in accord with studies of hepatocytes isolated from the *Lipn1*-deficiency *fld *(fatty liver lipodystrophy) mice, in which the rate of TAG synthesis was not changed in the hepatocytes isolated from adult mice, however, the rate of TAG synthesis increased in the hepatocytes isolated from 14-day-old *fld *mice. Adenovirus mediated Lipin-1 overexpression in the hepatocytes isolated from adult *fld *mice resulted in the suppression of VLDL-TAG secretion [92]. Moreover, adenovirus-mediated lipin-1 overexpression in the obese, insulin-resistant UCP-DTA mice resulted in decreased the fasting plasma TAG concentration [92]. The reason for the discrepancy between these studies regarding the role of lipin-1 in VLDL secretion remains to be explained. It has been shown that compartmentalization of lipin-1, in addition to its PAP1 activity, also plays a role in VLDL assembly and secretion [91]. Expression of a mutant form of lipin-1α, in which the nuclear localization signal sequence was removed, resulted in cytosolic presentation of the protein and diminished stimulation of VLDL_1 _secretion [91]. Nuclear localization of lipin-1 is probably important for transactivation of gene expression, as lipin-1 is known to act as a transcription activator [93]. The dual function of lipin-1 and its variable subcellular compartmentalization with respect to VLDL assembly and secretion requires further investigation.

The final step in the *de novo *synthesis of TAG is catalyzed by acyl-CoA:diacylglycerol acyltransferase (DGAT). Two *DGAT *genes encode the respective DGAT1 and DGAT2, which are structurally unrelated and show hepatic expression [94,95]. The DGAT1 knockout mouse has a normal fasting plasma TAG level despite reduced hepatic TAG [96]. Adenovirus mediated expression of DGAT1 in mice increased TAG in the liver but did not increase VLDL production [97]. However, overexpression of human DGAT1 in McA-RH7777 cells resulted in increased synthesis, cellular accumulation, and secretion of TAG as VLDL [98,99]. Concomitantly, intracellular degradation of apoB was decreased in DGAT1 overexpressing cells [98]. Overexpression of DGAT2 in McA-RH7777 cells also resulted in increased secretion of TAG and apoB [98], whereas knockdown of DGAT2 in mice with antisense oligonucleotide decreased TAG and apoB secretion as VLDL [100]. These studies suggest that although TAG synthesis is important for promoting VLDL production, the increased hepatic TAG may be compartmentalized and thus not necessarily be available for VLDL assembly or secretion.

Hepatic VLDL assembly and secretion is also profoundly influenced by alterations in the *de novo *biosynthesis of phospholipids, such as PE and PC. Two pathways are involved in hepatic PC synthesis; the CDP-choline pathway contributes approximately 70% of total hepatic PC synthesis, whereas the remainder 30% is synthesized through the PE methylation pathway. The PE methylation pathway is catalyzed by PE *N*-methyltransferase (PEMT), a product of the *Pemt2 *gene. The *Pemt2*^-/- ^mouse had normal liver PC content as a result of compensatory upregulation of CTP:phosphocholine cytidylyltransferase (CCT) activity; hence these mice did not display abnormal plasma lipid levels compared to control mice [101]. It appears that as long as CCT activity is intact, hepatic PC synthesis is uncompromised when the PEMT activity is absent. Lack of PEMT in the *Ldlr*^-/- ^background, however, resulted in a reduction of atherosclerosis incidence when the mice were fed with a high fat diet for 16 weeks [102]. These reduced atherosclerotic lesion development was attributed to lowered phospholipid content in apoB-containing lipoproteins (*e.g. *VLDL, IDL, and LDL) and reduced secretion of VLDL [102].

The rate limiting step in the CDP-choline pathway for PC synthesis is catalyzed by CCT. At least three CCT isoforms are known in mammals and they are encoded by two genes, namely *Pcyt1α *for CCTα and *Pcyt1b *for differentially spliced transcripts CCTβ2 and CCTβ3 [103]. It has been shown that plasma levels of HDL and VLDL were markedly reduced in mice lacking hepatic CCTα expression [104], indicating that the activity of hepatic CCT is an important determinant of VLDL and HDL *in vivo*. However, reconstituted CCTα in the knock-out hepatocytes by *ex vivo *infection with adenovirus encoding CCTα failed to stimulate VLDL secretion even though cellular PC levels returned to normal [105]. In contrary, adenoviral delivery of CCTα into knock-out mice normalized plasma HDL and VLDL [105]. The CCTα activity is thus required for lipoprotein metabolism *in vivo*.

Hepatic PE synthesis is likewise achieved through the CDP-ethanolamine pathway, in which CTP:phosphoethanolamine cytidylyltransferase (encoded by *Pcyt2*) catalyzes the rate regulatory step for the formation of CDP-ethanolamine [106]. Reduced CDP-ethanolamine synthesis in *Pcyt2*^+/- ^mice resulted in elevated plasma VLDL (at 32-36 weeks old), as well as progressive development of hepatosteatosis, obesity, and insulin resistance [107]. The increased hepatic TAG synthesis and secretion in *Pcyt2*^+/- ^mice are probably attributable to increased availability of diacylglycerol (as a result of limited CDP-ethanolamine) [107].

Because phospholipid synthesis not only supplies lipid substrates for VLDL but also contributes to the biogenesis and maintenance of ER/Golgi membranes, it impacts both the cargo and trafficking machinery for VLDL assembly and secretion. Studies with genetically modified mice lacking both PEMT and multiple drug-resistant protein 2 have suggested that the PE/PC ratio is a key regulator of cell membrane integrity in the liver and plays a role in the progression of steatosis into steatohepatitis [108]. It remains to be determined whether or not the composition of phospholipid species in the ER/Golgi membranes affects VLDL assembly/secretion.

## VLDL assembly and secretion influenced by vesicular trafficking factors

Protein factors involved in intracellular vesicular trafficking, primarily the small GTPase proteins, play a profound role in VLDL assembly and secretion. The requirement of COPII coated vesicles for apoB exiting from the ER has been demonstrated by an *in vitro *ER budding assay [109]. Formation of COPII coated vesicles is initiated by the GTPase Sar1 protein. Deficiency of Sar1 has been linked to intestinal apoB-lipoprotein secretion disorder known as chylomicron retention disease in humans [110]. Expression of a GDP-restricted mutant (T39N) of the Sar1 protein in McA-RH7777 cells prevented ER exit of apoB [109]. The anterograde transport from the Golgi apparatus is driven by the small GTPase, ADP ribosylation factor 1 (ARF1). Expression of a dominant-negative T31N mutant of ARF1 in McA-RH7777 cells resulted 80% reduction in the assembly of apoB-100 VLDL_1 _along with the loss of COPI from the Golgi apparatus. Overexpression of ARF1 in the cells resulted in an oleate dose dependent increase in VLDL_1 _secretion with a concomitant decrease in VLDL_2 _secretion [111].

The activities of two phospholipases have been shown to play a role in VLDL maturation, the calcium independent phospholipase A_2 _(iPLA_2_β) and phospholipase D (PLD_1_) [71,72,112]. The iPLA_2_β is an intracellular protein that does not have a Ca^2+^-dependent lipid-binding domain but contains ankyrin repeats that may mediate membrane binding. Inhibition of iPLA_2_β with chemical inhibitors or antisense RNA interfered with the formation of TAG-rich VLDL_1 _but not dense VLDL particles such as VLDL_2 _[71,72]. The PLD_1 _catalyzes the hydrolysis of PC to produce PA and choline and is activated by ARF1. Inactivation of PLD_1 _activity (and thus the formation of PA) in cultured hepatic cells using chemical inhibitors also blocked VLDL formation [71,72]. Likewise, expression of aberrant ARF1 that lost PLD_1_-activation function [111] or treatment with brefeldin A (an inhibitor of ARF1) [56,113] effectively prevented TAG-rich VLDL secretion with little effect on the secretion of denser particles. These results suggest that maturation of VLDL probably not only requires sufficient lipid substrate availability, but also depends upon the protein factors that effectively mediate the fusion between lipid droplets and apoB to form TAG-rich VLDL. The fusion events presumably require coordinated synthesis and fusion of vesicle themselves, as well as synthesis and fusion of cargo (*i.e. *lipid droplets and VLDL precursors) within the vesicles. Factors within the microsomal lumen that are responsible for cargo fusion during the final step of VLDL_1 _maturation remain to be determined.

## Cytosolic lipid droplet-associated proteins affecting VLDL assembly and secretion

Multiple protein factors that are found in association with cytosolic lipid droplets also influence VLDL assembly and secretion. Adipocyte differentiation-related protein (ADRP) is the major protein associated with cytosolic lipid droplets. Overexpression of ADRP in McA-RH7777 cells resulted in increased accumulation of cytosolic lipid droplets and a corresponding decrease in VLDL secretion [114]. Although the molecular mechanism responsible for the inhibitory effect of ADRP expression on VLDL secretion remains to be defined, it is possible that enlargement of cytosolic lipid droplets may diminish the microsomal TAG pool for VLDL secretion.

Recently, another lipid droplet associated protein CideB has been suggested to play a role in VLDL assembly and secretion [115]. CideB is a member of Cide (cell death-inducing DFFA45 (DNA fragmentation factor 45)-like effector) family that also includes CideA, and CideC (or Fsp27). While CideA is expressed at high levels in brown adipose tissue, CideB mRNA and proteins are detected in various tissues with the highest level of expression in the liver. Hepatic CideB exists as a smooth ER- and lipid droplet-associated protein. Mice deficient in CideA or CideB are resistant to high-fat diet-induced obesity and diabetes [116]. The role of CideB in regulating lipid homeostasis has been studied with the *Cideb*^-/- ^mice. Compared with wildtype littermates, the *Cideb*^-/- ^mice exhibited an increase in hepatic TAG content and reduced VLDL secretion [115]. Yeast two hybrid and co-immunoprecipitation experiments have shown a physical interaction between CideB and apoB [115]. These data suggest that the cytosolic lipids droplets are functionally in close contact with the ER where initial VLDL assembly takes place.

## Non-apoB apolipoproteins affecting VLDL assembly and secretion

In addition to apoB-100, hepatic VLDL particles also contain other apolipoproteins such as apoE and apoC. ApoE (299 amino acids) is a major protein constituent of TAG-rich lipoproteins including VLDL and chylomicrons. Experimental evidences obtained by several laboratories working with apoE overexpressing transgenic mice [117-119] or McA-RH7777 cells [120] have suggested that apoE plays a role in the formation of fully lipidated VLDL. The underlying mechanisms by which apoE expression promotes VLDL assembly and secretion were unclear. Recently, a detailed analysis conducted using McA-RH7777 cells treated with apoE specific siRNA or primary hepatocytes isolated from *apoE*^-/- ^mice have shown that the assembly (within the Golgi apparatus) or secretion of VLDL was independent of apoE expression [121]. Thus, the VLDL-associated apoE in the Golgi apparatus and media may not drive the formation of fully lipidated VLDL.

Apolipoprotein C-III is a small (79 amino acids) exchangeable apolipoprotein composed of multiple amphipathic α-helices, and is expressed mainly in the liver [122]. It has been reported recently that overexpression of human apoC-III in McA-RH7777 cells resulted in the overproduction and secretion of VLDL-TAG and VLDL-apoB under lipid rich conditions [76]. Overexpression of apoC-III also resulted in increased activity and expression of MTP. The ability of apoC-III to stimulate hepatic VLDL assembly and secretion was abolished by a naturally occurring mutation Ala23Thr [123] that was identified in human subjects with hypotriglyceridemia [124]. Thus apoC-III, a component of VLDL and HDL, appears to play an intracellular role in stimulating VLDL assembly and secretion [76,123]. Mechanisms by which apoC-III exerts the stimulatory effect on VLDL assembly and secretion are unclear.

The human *APOA5 *gene is a part of the apolipoprotein gene cluster that contains *APOA1, APOC3*, and *APOA4 *on chromosome 11 (11q23) [125]. Initial studies has revealed association of single nucleotide polymorphisms within the *APOA5 *locus with plasma TAG and VLDL in humans, and the effect is not related to the neighboring *APOC3 *gene markers [125]. Mice expressing the *APOA5 *transgene displayed a 65% decrease in plasma TAG levels; whereas *apoa5 *knock-out mice showed a 4-fold increase in plasma TAG concentration [125]. Thus, apoA-V (343 amino acids) has been viewed as a candidate gene regulating plasma TAG concentrations [126]. Overexpression of murine apoA-V in C57Bl/6 mice through adenovirus-mediated gene transfer decreased VLDL production rate in a dose-dependent manner by impairing apoB lipidation [127]. In the same mouse model, overexpression of apoA-V also resulted in decreased plasma TAG by enhancing lipoprotein lipase-mediated clearance of TAG-rich lipoproteins [127]. Transfection studies showed that recombinant apoA-V expressed in McA-RH7777 cells was unexpectedly associated with cytosolic lipid droplets, despite the fact that apoA-V possesses the signal peptide and is a secretory protein [128]. The mechanisms whereby apoA-V expression attenuates VLDL production remain to be defined.

## Lipoprotein receptors affecting VLDL assembly and secretion

Intracellular degradation of newly synthesized apoB-100 diminishes overall assembly and secretion of VLDL. The LDL receptor, a ubiquitously expressed protein responsible for the clearance of cholesterol-rich lipoproteins from blood stream through its ligands apoB-100 and apoE, promotes intracellular degradation of apoB-100 resulting in decreased VLDL secretion [129]. The loss of LDL receptor activity in the liver cells leads to increased secretion of VLDL particles, and the particles secreted are small with reduced TAG content [130,131]. Thus, the LDL receptor mediated apoB-100 degradation appears to preferentially target underlipidated particles. Mechanisms responsible for the LDL receptor mediated apoB-100 degradation may involve (i) rapid reuptake of nascent VLDL particles on the cell surface [129,132] and (ii) intracellular targeting of nascent VLDL particles to degradation [129,133]. Both mechanisms appear to require exit of apoB-100 from the ER and interaction of apoE or apoB-100 with the LDL receptor [134].

Expression and function of LDL receptor are negatively regulated by proprotein convertase subtilisin kexin type 9 (PCSK9) [135,136]. Attempts were made to determine whether the level of PCSK9 expression would affect apoB secretion or intracellular degradation. Transfection studies with McA-RH7777 cells that stably expressed the D374Y mutant form of PCSK9 (identified in human familial hypercholesterolemia) showed that the pathogenic variant expression resulted in increased secretion of apoB-100 lipoproteins (by 2-4-fold) but expression of the wildtype PCSK9 did not increase apoB-100 secretion [137]. These results might be interpreted as reduced degradation of nascent apoB-100 protein mediated by the LDL receptor. Lack of an effect of wildtype PCSK9 expression on apoB-100 secretion was also observed in transfection studies with the human hepatoma HuH7 cells [138]. Likewise, secretion of apoB-100 from primary hepatocytes of wildtype or *Pcsk9*^-/- ^mice was not significantly different [139]. The possible link between PCSK9, LDL receptor, and hepatic apoB-100 secretion remains to be determined.

Genetic absence of functional ATP binding cassette transporter A1 (ABCA1) in Tangier disease is associated with severely lowered plasma HDL and concomitantly elevated plasma TAG concentrations [140]. This metabolic abnormality has been recapitulated recently in McA-RH7777 cells where the ABCA1 was silenced using siRNA [141]. Unexpectedly, supplementation of culture media of the ABCA1 knockdown cells with nascent large HDL decreased the secretion of buoyant VLDL_1 _particles [141]. It appears that the large HDL, assembled by hepatic ABCA1, can attenuate VLDL secretion through the phosphoinositide 3 kinase (PI3K)-dependent signaling pathway [141]. This study is reminiscent of a previous observation that the PI3K activity is required for insulin-dependent inhibition of apoB secretion from primary rat hepatocytes [142].

Scavenger receptor B1 (SR-B1) is a receptor for selective uptake of HDL cholesterol and is also known to mediate the catabolism of apoB-containing lipoproteins. Overexpression of SR-B1 in mice via adenovirus-mediated infection resulted in increased plasma concentrations of VLDL-TAG and VLDL-apoB [143]. On the other hand, disruption of the *Scarb1 *gene expression in mice resulted in reduced VLDL production as well as MTP activity [143], suggesting that hepatic SR-B1 expression is closely linked to VLDL production.

Overall, the involvement of various lipoprotein receptors in regulating VLDL assembly and secretion has gradually been revealed, which introduces another level of complexity to the regulation of hepatic TAG homeostasis. Because these lipoprotein receptors are intimately involved in the metabolism of cholesterol (and phospholipids as well), such as uptake, efflux, and intracellular trafficking, it is possible that hepatic VLDL assembly and secretion are influenced by cellular cholesterol metabolism and related signaling events. The interrelationship between TAG and cholesterol metabolism and its link to VLDL assembly and secretion merits further investigation.

## Altered insulin and leptin signaling affecting VLDL assembly and secretion

Hepatic VLDL overproduction and impairment in catabolism/clearance of TAG-rich lipoproteins from circulation represent the two major contributors to hypertriglyceridemia. Many patients with hypertriglyceridemia manifest elevated plasma TAG, accumulation of small dense LDL particles and reduced HDL cholesterol particles, all of which are closely associated with cardiovascular diseases [144]. The other traits associated with hypertriglyceridemia include visceral obesity and insulin resistance, which further exacerbate the aberrant overproduction of VLDL as a result of excess flux of fatty acids (derived partly from lipolysis of plasma TAG-rich lipoproteins) into the liver. Overall, insulin treatment decreases hepatic VLDL production by limiting fatty acid influx into the liver, decreasing the stability of apoB, and promoting the posttranslational degradation of apoB, a process mediated through the PI3-K pathway [145]. Overproduction of hepatic VLDL that results from the loss of insulin responsiveness is often seen in insulin resistance conditions, which is associated with increased posttranslational stability of apoB-100 [145]. The insensitivity of liver cells to insulin mediated suppression of VLDL assembly is observed in fructose-induced insulin resistant hamster model [146], where insulin resistance is accompanied with hepatic inflammation. The fructose-induced, insulin-resistant hamsters exhibited reduced levels of the inhibitor of nuclear factor-κB (IκB) which resulted in a concomitant activation of the inflammatory nuclear factor-κB (NF-κB) cascade. Inhibition of NF-κB cascade with chemical inhibitors also resulted in decreased synthesis of apoB-100 in primary hepatocytes and HepG2 cells, and was probably attributable to the activation of insulin signaling and enhanced proteasomal degradation of apoB-100 [147]. On the other hand, activation of the NF-κB pathway, via adenovirus-mediated IκB kinase overexpression, resulted in increased apoB-100 synthesis as a result of suppressed insulin signaling through the NF-κB pathway [147]. Thus, there is an important link between the inflammatory IκB kinase-NF-κB signaling cascade, insulin signaling, and hepatic apoB100 synthesis and secretion.

The insensitivity of liver cells to insulin-mediated suppression of VLDL assembly is also observed in leptin-deficient obese mouse (*ob/ob*) [148]. Leptin is an adipose derived hormone that plays a key role in energy intake and expenditure. The leptin-deficient *ob/ob *mice and the leptin receptor-deficient *db/db *mice have been used extensively as model systems to study their roles in VLDL assembly and secretion [149]. The *in vivo *secretion rates of TAG and apoB were reduced in both male and female *ob/ob *mice as compared to their littermates. However, in *db/db *obese mice, only male mice showed reduced secretion of TAG. In *ob/ob *mice and *db/db *obese females, there was a small increase in apoB-100 secretion and no difference in apoB48 secretion. In these animals, the main cause of dyslipidemia was due to the impaired removal of VLDL from the circulation [149].

The *ob/ob *and *db/db *mice have been used to determine diet or protein factors that influence the pathophysiology of lipoprotein metabolism. For instance, the *db/db *mice have been crossed with mice expressing human cholesteryl ester transfer protein (CETP); the resulting mice displayed lowered levels of VLDL and LDL and became resistant to diet-induced atherosclerosis as compared to controls [150]. These results ascribe an anti-atherogenic role for CETP under diabetic obese conditions. The *ob/ob *mice crossed into the LDL receptor knockout background (*Ldlr*^-/-^) showed severe hyperlipidemia and spontaneous atherosclerosis, which was associated with increased hepatic TAG production, delayed VLDL clearance, and decreased hepatic uptake of LDL [151]. The *db/db *mice have also been used as a model to study diet-induced nonalcoholic steatohepatitis (NASH). Feeding *db/db *mice with methionine- and choline-deficient (MCD) diet induced liver injury [152,153], and were probably attributable to impaired hepatic secretion of VLDL [154]. The MCD-fed *db/db *mice developed hepatic steatosis and displayed insulin resistance that is severer than MCD-fed control mice [153]. These studies indicate that increased fatty acid uptake together with decreased secretion of VLDL represent the major insult that lead to hepatic TAG accumulation under MCD diet conditions.

Liver × receptor α (LXRα), the master regulator of lipid metabolism [155], also regulates hepatic VLDL production. Activation of LXR in hamsters fed with an LXR agonist resulted in markedly increased plasma TAG and VLDL and enhanced expression of sterol response element binding protein-1c (SREBP-1c) and its target lipogenesis genes including steroyl CoA desaturase and fatty acid synthase [156]. Furthermore, LXR activation also led to enhanced stability of newly synthesized apoB and increased secretion of TAG-rich VLDL-apoB. Increased stability of apoB-100 was probably achieved through attenuated insulin receptor and insulin receptor substrate-1 tyrosine phosphorylation and concomitant increases in protein tyrosine phosphatase 1B [156].

Studies with rats have shown that acute leptin treatment, like that of insulin, lowered plasma VLDL-associated TAG [157,158]. However, the molecular mechanism responsible for the leptin-induced hypotriglyceridemia may be distinct from that of insulin. The leptin treatment resulted in decreased lipogenesis and enhanced β-oxidation, thus limiting lipid substrate for VLDL assembly/secretion without affecting hepatic apoB levels [159,160]. On the other hand, insulin treatment had no effect on β-oxidation but decreased hepatic apoB levels [160], probably owning to enhanced posttranslational degradation of apoB. In summary, hepatic VLDL production is promoted under conditions where post-translational stability of apoB is augmented (*e.g. *enhanced lipid substrate supply), and its production is suppressed when post-translational degradation of apoB is increased (*e.g. *decreased lipid substrate availability).

## Intracellular degradation of newly synthesized apoB

Intracellular degradation of apoB refers to a process whereby the newly synthesized apoB proteins are degraded prior to secretion. It has been shown that apoB degradation occurs during and after the protein translation, and the co- and post-translational degradation of apoB takes place in both ER and post-ER compartments. Attenuated intracellular degradation of newly synthesized apoB is accompanied with increased VLDL production, as often observed under chronic hyperinsulinemia and insulin resistance conditions [161,162]. Under conditions unfavorable for apoB folding or lipid assembly, the newly synthesized apoB polypeptide undergoes ubiquitin-mediated proteasomal degradation [56,163-165]. Lack of sufficient lipid supply or availability often triggers the degradation process. Under certain conditions, apoB degradation occurs even after an assembly intermediate (*i.e. *a VLDL precursor) has already been assembled (for extensive review on apoB degradation see [166-169]).

Non-proteasomal degradation pathway for apoB has been described which involves autophagosomes [170,171]. Autophagosomes are membrane structures that encase and target intracellular substrates to lysosomes for disposal, a process termed autophagy [172]. The involvement of autophagy process for apoB degradation appears to be pronounced when ubiquitin/proteasome pathway is inhibited, and the apoB proteins accumulated in the crescent-shaped structures that are in close proximity to cytosolic lipid droplets [173]. It has been postulated that the crescent structures are the sites where proteasomal and autophagosomal pathways converge [173]. The autophagy mediated apoB degradation has also been suggested in cells treated with n-3 fatty acids such as docosahexaenoic acid (22:6 n-3), and under this condition apoB also undergoes aggregation and oxidation [78]. As mentioned earlier, the n-3 fatty acid induced intracellular apoB degradation is probably related to the poor utilization of TAG molecules for VLDL assembly and secretion [72]. Missense mutations within the βα1 domain of apoB also resulted in increased degradation through autophagy [50], which presumably resulted from impaired secretion of the mutant apoB as lipoproteins.

In summary, in addition to the ubiquitin/proteasome pathway presumably responsible for ER-associated degradation of misfolded apoB polypeptides, the autophagosome-mediated apoB and apoB-lipoprotein degradation may represent an alternative pathway (post-ER degradation) for the disposal of aborted assembly intermediates. Since augmented intracellular degradation of apoB and apoB-lipoproteins is a potential means to suppress overproduction of the atherogenic VLDL/LDL, further studies are merited to identify and characterize factors involved in autophagy-mediated apoB degradation.

## Concluding remarks and perspectives

**Figure 2:**
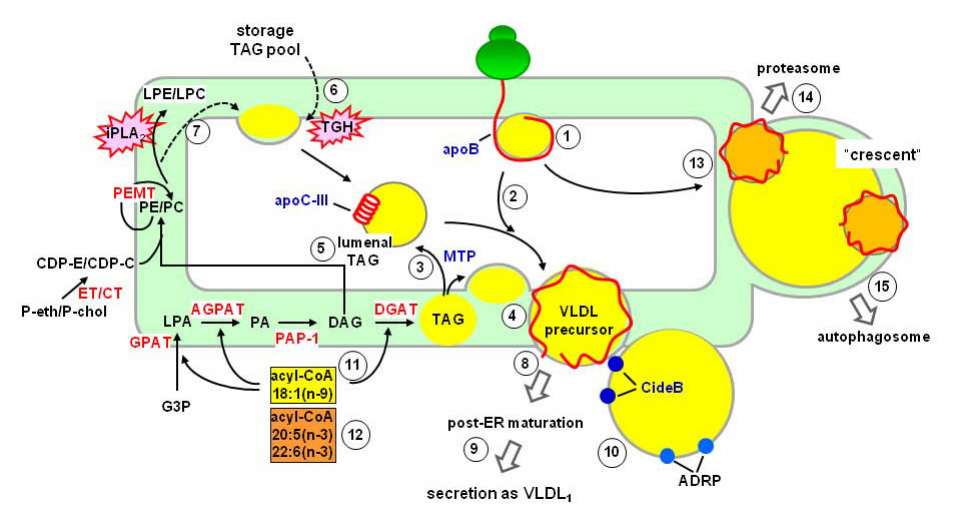
**Protein and lipid factors affecting VLDL assembly and secretion**. The apoB polypeptide initiates lipid recruitment during translation and translocation (1); this process may or may not require the activity of MTP. The nascent apoB-lipid particle acquires, in a step-wise fashion, additional TAG (2). The activity of MTP is required for partitioning of TAG into the lumen (3) or the membranes of ER microsomes for VLDL assembly (4). The lumenal TAG substrate, referred to as "lumenal lipid droplet", exists in association with non-apoB apolipoproteins such as apoC-III (5). In addition to that synthesized from the *de novo *pathway (catalyzed by GPAT, AGPAT, PAP-1, and DGAT), the TAG substrate utilized for VLDL assembly is also derived from esterification of fatty acyl chains originated from TGH-mediated hydrolysis of existing storage and lumenal TAG pools (6) or from phospholipid turnover catalyzed by iPLA2 (7). The resulting VLDL precursor exits the ER through coatomer-mediated budding/vesiculation events (8), and maturation of VLDL_1 _is achieved through ER/Golgi trafficking (9). Proteins associated with cytosolic lipid droplets, such as CideB and ADRP (10), influence VLDL assembly through an unknown mechanism. Molecular species of fatty acids have a profound impact on VLDL production; oleic acid (18:1 n-9) promotes (11) whereas EPA (20:5 n-3) and DHA (22:6 n-3) attenuate (12) VLDL assembly/secretion. Misfolded apoB protein and aborted lipoprotein assembly intermediates are targeted for intracellular degradation (13). Two degradative pathways, namely the ubiquitin/proteasome pathway (14) and autophagy (15), may involve formation of "crescent" structures that contain apoB and are in close association with cytosolic lipid droplets. *ADRP*, adipocyte differentiation-related protein; *AGPAT*, acylglycerol-3-phosphate acyltransferase; *CDP-C*, CDP-choline; *CDP-E*, CDP-ethanolamine; *CT*, CTP:phosphocholine cytidylyltransferase; *DAG*, diacylglycerol; *DGAT*, acyl-CoA:diacylglycerol acyltransferase; *ET*, CTP:phosphoethanolamine cytidylyltransferase; *G-3-P*, glycerol-3-phosphate; *GPAT*, glycerol-3-phosphate acyltransferase; *LPA*, lysophosphatidate; *LPC*, lysophophatidylcholine; *LPE*, lysophosphatidylethanolamine; *MTP*, microsomal triglyceride-transfer protein; *PAP-1*, phosphatidate phosphatase-1; *PC*, phosphatidylcholine; *P-cho*, phosphocholine; *PE*, phosphatidylethanolamine; *P-eth*, phosphoethanolamine; *TAG*, triacylglycerol; *VLDL*, very low density lipoproteins.

The past three decades have witnessed a tremendous advancement in our knowledge and understanding of protein and lipids factors that influence VLDL assembly and secretion. Amino acid sequences within apoB-100, particularly the N-terminal βα1 domain and the β-sheet enriched β1 domain, have been recognized as functional elements governing VLDL assembly/secretion. The role of MTP in facilitating lipid mobilization into the microsomal lumen and that of protein factors (*e.g. *apoC-III) in promoting bulk TAG incorporation during VLDL maturation is being revealed. In addition, enzymes that are directly involved in hepatic lipogenesis and glycerolipids biogenesis, such as lipin-1 and DGAT among others, have been cloned and characterized. Delineation and characterization of regulatory pathways of these lipid synthesis enzymes have provided molecular explanations for hormonal regulation (*e.g. *insulin and glucocorticoids) of VLDL assembly and secretion. Moreover, a comprehensive view of the interplay among various transcription factors (e.g. SREBP-1, LXR, PPAR, and PGC-1) in regulating hepatic lipogenesis, β-oxidation, and glycerolipids biogenesis has begun to emerge, and their implications to VLDL assembly/secretion under stress conditions require further investigation. Finally, new mechanisms such as proteasomes and autophagosome that may play a role in intracellular degradation of apoB thus attenuate hepatic VLDL production have been suggested and also merit additional studies. Various protein and lipid factors that participate and influence hepatic VLDL assembly/secretion are depicted in Fig. 2.

Other than understanding the biosynthesis of lipid substrates required for VLDL assembly and secretion, a great deal of knowledge has also accumulated with respect to the temporal and spatial events associated with lipid incorporation into VLDL. It is clear that VLDL precursors are assembled within the ER and the maturation of TAG-rich VLDL_1 _take place in post-ER compartments. However, several questions remain unanswered. For example, it is unclear about the biochemical origin(s) of TAG or the relative contribution between the *de novo *synthesis and the hydrolysis/re-esterification pathways during VLDL maturation under normal or stress conditions. It is also unclear what protein factors or membrane structures are required for the mobilization of lumenal TAG substrates or their delivery to VLDL precursors. Although increasing evidence suggests that protein factors associated with cytosolic lipid droplets (*e.g. *CideB and ADRP) may participate or influence lumenal VLDL assembly, the cellular mechanisms through which the *cytosolic *and *lumenal *components communicate remains an enigma. Finally, knowledge gained from cell culture and animal studies remains to be verified in humans with advanced *in vivo *technologies to validate the pathophysiological relevance.

## List of abbreviations used

ABCA1: ATP binding cassette transporter A1; ADRP: adipocyte differentiation-related protein; AGPAT: 1-acylglycerol-3-phosphate O-acyltransferase; ARF1: ADP ribosylation factor 1; CCT: CTP:phosphocholine cytidylyltransferase; CETP: cholesteryl ester transfer protein; DGAT: acyl-CoA:diacylglycerol acyltransferase; DHA: docosahexaenoic acid; EPA: eicosapentaenoic acid; ER: endoplasmic reticulum; FHBL: familial hypobetalipoproteinemia; GPAT: glycerol-3-phosphate acyltransferase; HDL: high density lipoproteins; IDL: intermediate density lipoproteins; IκB: inhibitor of nuclear factor-κB; LDL: low density lipoproteins; L-FABP: liver fatty acid binding protein; LPA: lysophosphatidate; LXRα: liver × receptor α; MCD: methionine- and choline-deficient; MTP: microsomal triglyceride-transfer protein; NF-κB: nuclear factor-κB; PA: phosphatidate; PAP-1: phosphatidate phosphatase-1; PC: phosphatidylcholine; PCSK9: proprotein convertase subtilisin kexin type 9; PDI: protein disulfide isomerase; PE: phosphatidylethanolamine; PEMT: PE *N*-methyltransferase; PGC: peroxisome proliferator-activated receptor gamma coactivator; PI3K: phosphoinositide 3 kinase; PPAR: peroxisome proliferator-activated receptor; SHP: small heterodimer partner; SR-B1: scavenger receptor B1; SREBP: sterol regulatory element binding protein; TAG: triacylglycerol; TGH: triacylglycerol hydrolase; VLDL: very low density lipoproteins.

## Competing interests

The authors declare that they have no competing interests.

## Authors' contributions

Both MS and ZY drafted, edited, and final approved the manuscript.

## Authors' information

Meenakshi Sundaram, PhD, Research Associate, Department of Biochemistry, Microbiology and Immunology, University of Ottawa

Zemin Yao, PhD, Professor and Chair, Department of Biochemistry, Microbiology and Immunology, Ottawa Institute of Systems Biology, University of Ottawa

## References

[B1] Law SW Lackner KJ Hospattankar AV Anchors JM Sakaguchi AY Naylor SL Brewer HB Jr Human apolipoprotein B-100: cloning, analysis of liver mRNA, and assignment of the gene to chromosome 2 Proc Natl Acad Sci USA 1985 82 8340 8344 10.1073/pnas.82.24.8340 3001697 PMC390911

[B2] Lusis AJ West R Mehrabian M Reuben MA LeBoeuf RC Kaptein JS Johnson DF Schumaker VN Yuhasz MP Schotz MC Cloning and expression of apolipoprotein B, the major protein of low and very low density lipoproteins Proc Natl Acad Sci USA 1985 82 4597 4601 10.1073/pnas.82.14.4597 3860811 PMC390432

[B3] Deeb SS Disteche C Motulsky AG Lebo RV Kan YW Chromosomal localization of the human apolipoprotein B gene and detection of homologous RNA in monkey intestine Proc Natl Acad Sci USA 1986 83 419 422 10.1073/pnas.83.2.419 3455779 PMC322870

[B4] Chen SH Habib G Yang CY Gu ZW Lee BR Weng SA Silberman SR Cai SJ Deslypere JP Rosseneu M Apolipoprotein B-48 is the product of a messenger RNA with an organ-specific in-frame stop codon Science 1987 238 363 366 10.1126/science.3659919 3659919

[B5] Kane JP Hardman DA Paulus HE Heterogeneity of apolipoprotein B: isolation of a new species from human chylomicrons Proc Natl Acad Sci USA 1980 77 2465 2469 10.1073/pnas.77.5.2465 6930644 PMC349420

[B6] Tennyson GE Sabatos CA Higuchi K Meglin N Brewer HB Jr Expression of apolipoprotein B mRNAs encoding higher- and lower-molecular weight isoproteins in rat liver and intestine Proc Natl Acad Sci USA 1989 86 500 504 10.1073/pnas.86.2.500 2911593 PMC286498

[B7] Segrest JP Jones MK De LH Dashti N Structure of apolipoprotein B-100 in low density lipoproteins J Lipid Res 2001 42 1346 1367 11518754

[B8] Mann CJ Anderson TA Read J Chester SA Harrison GB Kochl S Ritchie PJ Bradbury P Hussain FS Amey J The structure of vitellogenin provides a molecular model for the assembly and secretion of atherogenic lipoproteins J Mol Biol 1999 285 391 408 10.1006/jmbi.1998.2298 9878414

[B9] Jiang ZG Gantz D Bullitt E McKnight CJ Defining lipid-interacting domains in the N-terminal region of apolipoprotein B Biochemistry 2006 45 11799 11808 10.1021/bi060600w 17002280 PMC2519233

[B10] Dashti N Gandhi M Liu X Lin X Segrest JP The N-terminal 1000 residues of apolipoprotein B associate with microsomal triglyceride transfer protein to create a lipid transfer pocket required for lipoprotein assembly Biochemistry 2002 41 6978 6987 10.1021/bi011757l 12033930

[B11] Jiang ZG Simon MN Wall JS McKnight CJ Structural analysis of reconstituted lipoproteins containing the N-terminal domain of apolipoprotein B Biophys J 2007 92 4097 4108 10.1529/biophysj.106.101105 17369413 PMC1868998

[B12] Ren G Rudenko G Ludtke SJ Deisenhofer J Chiu W Pownall HJ Model of human low-density lipoprotein and bound receptor based on cryoEM Proc Natl Acad Sci USA 2010 107 1059 1064 10.1073/pnas.0908004107 20080547 PMC2798884

[B13] Hussain MM Bakillah A Nayak N Shelness GS Amino acids 430-570 in apolipoprotein B are critical for its binding to microsomal triglyceride transfer protein J Biol Chem 1998 273 25612 25615 10.1074/jbc.273.40.25612 9748226

[B14] Segrest JP Jones MK Dashti N N-terminal domain of apolipoprotein B has structural homology to lipovitellin and microsomal triglyceride transfer protein: a "lipid pocket" model for self-assembly of apob-containing lipoprotein particles J Lipid Res 1999 40 1401 1416 10428976

[B15] Liang J Ginsberg HN Microsomal triglyceride transfer protein binding and lipid transfer activities are independent of each other, but both are required for secretion of apolipoprotein B lipoproteins from liver cells J Biol Chem 2001 276 28606 28612 10.1074/jbc.M100294200 11358959

[B16] Bradbury P Mann CJ Kochl S Anderson TA Chester SA Hancock JM Ritchie PJ Amey J Harrison GB Levitt DG A common binding site on the microsomal triglyceride transfer protein for apolipoprotein B and protein disulfide isomerase J Biol Chem 1999 274 3159 3164 10.1074/jbc.274.5.3159 9915855

[B17] Hussain MM Bakillah A Jamil H Apolipoprotein B binding to microsomal triglyceride transfer protein decreases with increases in length and lipidation: implications in lipoprotein biosynthesis Biochemistry 1997 36 13060 13067 10.1021/bi971395a 9335568

[B18] Kreuzer J White AL Knott TJ Jien ML Mehrabian M Scott J Young SG Haberland ME Amino terminus of apolipoprotein B suffices to produce recognition of malondialdehyde-modified low density lipoprotein by the scavenger receptor of human monocyte-macrophages J Lipid Res 1997 38 324 342 9162752

[B19] Sivaram P Choi SY Curtiss LK Goldberg IJ An amino-terminal fragment of apolipoprotein B binds to lipoprotein lipase and may facilitate its binding to endothelial cells J Biol Chem 1994 269 9409 9412 8144523

[B20] Shelness GS Hou L Ledford AS Parks JS Weinberg RB Identification of the lipoprotein initiating domain of apolipoprotein B J Biol Chem 2003 278 44702 44707 10.1074/jbc.M307562200 12941937

[B21] Wang L Martin DD Genter E Wang J McLeod RS Small DM Surface study of apoB1694-1880, a sequence that can anchor apoB to lipoproteins and make it nonexchangeable J Lipid Res 2009 50 1340 1352 10.1194/jlr.M900040-JLR200 19251580 PMC2694333

[B22] Hospattankar AV Law SW Lackner K Brewer HB Jr Identification of low density lipoprotein receptor binding domains of human apolipoprotein B-100: a proposed consensus LDL receptor binding sequence of apoB-100 Biochem Biophys Res Commun 1986 139 1078 1085 10.1016/S0006-291X(86)80287-X 3767991

[B23] Yang CY Chen SH Gianturco SH Bradley WA Sparrow JT Tanimura M Li WH Sparrow DA DeLoof H Rosseneu M Sequence, structure, receptor-binding domains and internal repeats of human apolipoprotein B-100 Nature 1986 323 738 742 10.1038/323738a0 3095664

[B24] Wang L Walsh MT Small DM Apolipoprotein B is conformationally flexible but anchored at a triolein/water interface: a possible model for lipoprotein surfaces Proc Natl Acad Sci USA 2006 103 6871 6876 10.1073/pnas.0602213103 16636271 PMC1458986

[B25] Gretch DG Sturley SL Wang L Lipton BA Dunning A Grunwald KA Wetterau JR Yao Z Talmud P Attie AD The amino terminus of apolipoprotein B is necessary but not sufficient for microsomal triglyceride transfer protein responsiveness J Biol Chem 1996 271 8682 8691 10.1074/jbc.271.15.8682 8621500

[B26] McLeod RS Wang Y Wang S Rusinol A Links P Yao Z Apolipoprotein B sequence requirements for hepatic very low density lipoprotein assembly. Evidence that hydrophobic sequences within apolipoprotein B48 mediate lipid recruitment J Biol Chem 1996 271 18445 18455 10.1074/jbc.271.24.14124 8702489

[B27] Liang J Wu X Jiang H Zhou M Yang H Angkeow P Huang LS Sturley SL Ginsberg H Translocation efficiency, susceptibility to proteasomal degradation, and lipid responsiveness of apolipoprotein B are determined by the presence of beta sheet domains J Biol Chem 1998 273 35216 35221 10.1074/jbc.273.52.35216 9857060

[B28] Tran K Boren J Macri J Wang Y McLeod R Avramoglu RK Adeli K Yao Z Functional analysis of disulfide linkages clustered within the amino terminus of human apolipoprotein B J Biol Chem 1998 273 7244 7251 10.1074/jbc.273.13.7244 9516417

[B29] Huang XF Shelness GS Identification of cysteine pairs within the amino-terminal 5% of apolipoprotein B essential for hepatic lipoprotein assembly and secretion J Biol Chem 1997 272 31872 31876 10.1074/jbc.272.50.31872 9395534

[B30] Ingram MF Shelness GS Apolipoprotein B-100 destined for lipoprotein assembly and intracellular degradation undergoes efficient translocation across the endoplasmic reticulum membrane J Lipid Res 1996 37 2202 2214 8906597

[B31] Taniguchi T Ishikawa Y Tsunemitsu M Fukuzaki H The structures of the asparagine-linked sugar chains of human apolipoprotein B-100 Arch Biochem Biophys 1989 273 197 205 10.1016/0003-9861(89)90179-3 2757392

[B32] Vukmirica J Nishimaki-Mogami T Tran K Shan J McLeod RS Yuan J Yao Z The N-linked oligosaccharides at the amino terminus of human apoB are important for the assembly and secretion of VLDL J Lipid Res 2002 43 1496 1507 10.1194/jlr.M200077-JLR200 12235182

[B33] Dunphy JT Linder ME Signalling functions of protein palmitoylation Biochim Biophys Acta 1998 1436 245 261 9838145 10.1016/s0005-2760(98)00130-1

[B34] Hoeg JM Meng MS Ronan R Demosky SJ Jr Fairwell T Brewer HB Jr Apolipoprotein B synthesized by Hep G2 cells undergoes fatty acid acylation J Lipid Res 1988 29 1215 1220 2846736

[B35] Zhao Y McCabe JB Vance J Berthiaume LG Palmitoylation of apolipoprotein B is required for proper intracellular sorting and transport of cholesteroyl esters and triglycerides Mol Biol Cell 2000 11 721 734 10679026 10.1091/mbc.11.2.721 PMC14805

[B36] Vukmirica J Tran K Liang X Shan J Yuan J Miskie BA Hegele RA Resh MD Yao Z Assembly and secretion of very low density lipoproteins containing apolipoprotein B48 in transfected McA-RH7777 cells. Lack of evidence that palmitoylation of apolipoprotein B48 is required for lipoprotein secretion J Biol Chem 2003 278 14153 14161 10.1074/jbc.M211995200 12582154

[B37] Schonfeld G Familial hypobetalipoproteinemia: a review J Lipid Res 2003 44 878 883 10.1194/jlr.R300002-JLR200 12639976

[B38] Heiss G Tamir I Davis CE Tyroler HA Rifkand BM Schonfeld G Jacobs D Frantz ID Jr Lipoprotein-cholesterol distributions in selected North American populations: the lipid research clinics program prevalence study Circulation 1980 61 302 315 7351055 10.1161/01.cir.61.2.302

[B39] McCormick SP Fellowes AP Walmsley TA George PM Apolipoprotein B-32: a new truncated mutant of human apolipoprotein B capable of forming particles in the low density lipoprotein range Biochim Biophys Acta 1992 1138 290 296 1562615 10.1016/0925-4439(92)90006-9

[B40] Wagner RD Krul ES Tang J Parhofer KG Garlock K Talmud P Schonfeld G ApoB-54.8, a truncated apolipoprotein found primarily in VLDL, is associated with a nonsense mutation in the apoB gene and hypobetalipoproteinemia J Lipid Res 1991 32 1001 1011 1940616

[B41] Pullinger CR Hillas E Hardman DA Chen GC Naya-Vigne JM Iwasa JA Hamilton RL Lalouel JM Williams RR Kane JP Two apolipoprotein B gene defects in a kindred with hypobetalipoproteinemia, one of which results in a truncated variant, apoB-61, in VLDL and LDL J Lipid Res 1992 33 699 710 1619363

[B42] Krul ES Parhofer KG Barrett PH Wagner RD Schonfeld G ApoB-75, a truncation of apolipoprotein B associated with familial hypobetalipoproteinemia: genetic and kinetic studies J Lipid Res 1992 33 1037 1050 1431583

[B43] Farese RV Jr Garg A Pierotti VR Vega GL Young SG A truncated species of apolipoprotein B, B-83, associated with hypobetalipoproteinemia J Lipid Res 1992 33 569 577 1527480

[B44] Fouchier SW Sankatsing RR Peter J Castillo S Pocovi M Alonso R Kastelein JJ Defesche JC High frequency of APOB gene mutations causing familial hypobetalipoproteinaemia in patients of Dutch and Spanish descent J Med Genet 2005 42 e23 10.1136/jmg.2004.029454 15805152 PMC1736043

[B45] Chen Z Fitzgerald RL Schonfeld G Hypobetalipoproteinemic mice with a targeted apolipoprotein (Apo) B-27.6-specifying mutation: in vivo evidence for an important role of amino acids 1254-1744 of ApoB in lipid transport and metabolism of the apoB-containing lipoprotein J Biol Chem 2002 277 14135 14145 11839763 10.1074/jbc.M200617200

[B46] McLeod RS Zhao Y Selby SL Westerlund J Yao Z Carboxyl-terminal truncation impairs lipid recruitment by apolipoprotein B100 but does not affect secretion of the truncated apolipoprotein B-containing lipoproteins J Biol Chem 1994 269 2852 2862 8300620

[B47] Burnett JR Shan J Miskie BA Whitfield AJ Yuan J Tran K McKnight CJ Hegele RA Yao Z A novel nontruncating APOB gene mutation, R463W, causes familial hypobetalipoproteinemia J Biol Chem 2003 278 13442 13452 10.1074/jbc.M300235200 12551903

[B48] Burnett JR Zhong S Jiang ZG Hooper AJ Fisher EA McLeod RS Zhao Y Barrett PH Hegele RA van Bockxmeer FM Missense mutations in APOB within the betaalpha1 domain of human APOB-100 result in impaired secretion of ApoB and ApoB-containing lipoproteins in familial hypobetalipoproteinemia J Biol Chem 2007 282 24270 24283 10.1074/jbc.M702442200 17588943

[B49] Tarugi P Averna M Di LE Cefalu AB Noto D Magnolo L Cattin L Bertolini S Calandra S Molecular diagnosis of hypobetalipoproteinemia: an ENID review Atherosclerosis 2007 195 e19 e27 10.1016/j.atherosclerosis.2007.05.003 17570373

[B50] Zhong S Magnolo AL Sundaram M Zhou H Yao EF Di LE Loria P Wang S Bamji-Mirza M Wang L Nonsynonymous mutations within APOB in human familial hypobetalipoproteinemia: evidence for feedback inhibition of lipogenesis and postendoplasmic reticulum degradation of apolipoprotein B J Biol Chem 2010 285 6453 6464 10.1074/jbc.M109.060467 20032471 PMC2825441

[B51] Jamil H Dickson JK Jr Chu CH Lago MW Rinehart JK Biller SA Gregg RE Wetterau JR Microsomal triglyceride transfer protein. Specificity of lipid binding and transport J Biol Chem 1995 270 6549 6554 10.1074/jbc.270.12.6549 7896791

[B52] Wetterau JR Combs KA Spinner SN Joiner BJ Protein disulfide isomerase is a component of the microsomal triglyceride transfer protein complex J Biol Chem 1990 265 9800 9807 2351674

[B53] Wang L Fast DG Attie AD The enzymatic and non-enzymatic roles of protein-disulfide isomerase in apolipoprotein B secretion J Biol Chem 1997 272 27644 27651 10.1074/jbc.272.44.27644 9346903

[B54] Wetterau JR Aggerbeck LP Bouma ME Eisenberg C Munck A Hermier M Schmitz J Gay G Rader DJ Gregg RE Absence of microsomal triglyceride transfer protein in individuals with abetalipoproteinemia Science 1992 258 999 1001 10.1126/science.1439810 1439810

[B55] Read J Anderson TA Ritchie PJ Vanloo B Amey J Levitt D Rosseneu M Scott J Shoulders CC A mechanism of membrane neutral lipid acquisition by the microsomal triglyceride transfer protein J Biol Chem 2000 275 30372 30377 10.1074/jbc.C000364200 10893406

[B56] Zhou M Fisher EA Ginsberg HN Regulated Co-translational ubiquitination of apolipoprotein B100. A new paradigm for proteasomal degradation of a secretory protein J Biol Chem 1998 273 24649 24653 10.1074/jbc.273.38.24649 9733761

[B57] Wang Y Tran K Yao Z The activity of microsomal triglyceride transfer protein is essential for accumulation of triglyceride within microsomes in McA-RH7777 cells. A unified model for the assembly of very low density lipoproteins J Biol Chem 1999 274 27793 27800 10.1074/jbc.274.39.27793 10488124

[B58] Rustaeus S Stillemark P Lindberg K Gordon D Olofsson SO The microsomal triglyceride transfer protein catalyzes the post-translational assembly of apolipoprotein B-100 very low density lipoprotein in McA-RH7777 cells J Biol Chem 1998 273 5196 5203 10.1074/jbc.273.9.5196 9478974

[B59] Gordon DA Jamil H Progress towards understanding the role of microsomal triglyceride transfer protein in apolipoprotein-B lipoprotein assembly Biochim Biophys Acta 2000 1486 72 83 10856714 10.1016/s1388-1981(00)00049-4

[B60] Wang Y McLeod RS Yao Z Normal activity of microsomal triglyceride transfer protein is required for the oleate-induced secretion of very low density lipoproteins containing apolipoprotein B from McA-RH7777 cells J Biol Chem 1997 272 12272 12278 10.1074/jbc.272.19.12272 9139669

[B61] Kulinski A Rustaeus S Vance JE Microsomal triacylglycerol transfer protein is required for lumenal accretion of triacylglycerol not associated with ApoB, as well as for ApoB lipidation J Biol Chem 2002 277 31516 31525 10.1074/jbc.M202015200 12072432

[B62] Hebbachi A Gibbons GF Inactivation of microsomal triglyceride transfer protein impairs the normal redistribution but not the turnover of newly synthesized glycerolipid in the cytosol, endoplasmic reticulum and Golgi of primary rat hepatocytes Biochim Biophys Acta 1999 1441 36 50 10526226 10.1016/s1388-1981(99)00138-9

[B63] Chang BH Liao W Li L Nakamuta M Mack D Chan L Liver-specific inactivation of the abetalipoproteinemia gene completely abrogates very low density lipoprotein/low density lipoprotein production in a viable conditional knockout mouse J Biol Chem 1999 274 6051 6055 10.1074/jbc.274.10.6051 10037685

[B64] Raabe M Veniant MM Sullivan MA Zlot CH Bjorkegren J Nielsen LB Wong JS Hamilton RL Young SG Analysis of the role of microsomal triglyceride transfer protein in the liver of tissue-specific knockout mice J Clin Invest 1999 103 1287 1298 10.1172/JCI6576 10225972 PMC408359

[B65] Spann NJ Kang S Li AC Chen AZ Newberry EP Davidson NO Hui ST Davis RA Coordinate transcriptional repression of liver fatty acid-binding protein and microsomal triglyceride transfer protein blocks hepatic very low density lipoprotein secretion without hepatosteatosis J Biol Chem 2006 281 33066 33077 10.1074/jbc.M607148200 16950764

[B66] Huang J Iqbal J Saha PK Liu J Chan L Hussain MM Moore DD Wang L Molecular characterization of the role of orphan receptor small heterodimer partner in development of fatty liver Hepatology 2007 46 147 157 10.1002/hep.21632 17526026

[B67] Hribal ML Nakae J Kitamura T Shutter JR Accili D Regulation of insulin-like growth factor-dependent myoblast differentiation by Foxo forkhead transcription factors J Cell Biol 2003 162 535 541 10.1083/jcb.200212107 12925703 PMC2173790

[B68] Kamagate A Qu S Perdomo G Su D Kim DH Slusher S Meseck M Dong HH FoxO1 mediates insulin-dependent regulation of hepatic VLDL production in mice J Clin Invest 2008 118 2347 2364 18497885 10.1172/JCI32914 PMC2391277

[B69] Wolfrum C Stoffel M Coactivation of Foxa2 through Pgc-1beta promotes liver fatty acid oxidation and triglyceride/VLDL secretion Cell Metab 2006 3 99 110 10.1016/j.cmet.2006.01.001 16459311

[B70] Dolinsky VW Douglas DN Lehner R Vance DE Regulation of the enzymes of hepatic microsomal triacylglycerol lipolysis and re-esterification by the glucocorticoid dexamethasone Biochem J 2004 378 967 974 10.1042/BJ20031320 14662008 PMC1224021

[B71] Tran K Wang Y DeLong CJ Cui Z Yao Z The assembly of very low density lipoproteins in rat hepatoma McA-RH7777 cells is inhibited by phospholipase A2 antagonists J Biol Chem 2000 275 25023 25030 10.1074/jbc.M908971199 10827200

[B72] Tran K Sun F Cui Z Thorne-Tjomsland G St GC Lapierre LR McLeod RS Jamieson JC Yao Z Attenuated secretion of very low density lipoproteins from McA-RH7777 cells treated with eicosapentaenoic acid is associated with impaired utilization of triacylglycerol synthesized via phospholipid remodeling Biochim Biophys Acta 2006 1761 463 473 16675301 10.1016/j.bbalip.2006.03.018

[B73] Robichaud JC van d V Yao Z Trigatti B Vance DE Hepatic uptake and metabolism of phosphatidylcholine associated with high density lipoproteins Biochim Biophys Acta 2009 1790 538 551 19250958 10.1016/j.bbagen.2009.02.010

[B74] Sato K Cho Y Tachibana S Chiba T Schneider WJ Akiba Y Impairment of VLDL secretion by medium-chain fatty acids in chicken primary hepatocytes is affected by the chain length J Nutr 2005 135 1636 1641 15987842 10.1093/jn/135.7.1636

[B75] Tachibana S Sato K Cho Y Chiba T Schneider WJ Akiba Y Octanoate reduces very low-density lipoprotein secretion by decreasing the synthesis of apolipoprotein B in primary cultures of chicken hepatocytes Biochim Biophys Acta 2005 1737 36 43 16226916 10.1016/j.bbalip.2005.09.001

[B76] Sundaram M Zhong S Bou Khalil M Links PH Zhao Y Iqbal J Hussain MM Parks RJ Wang Y Yao Z Expression of apolipoprotein C-III in McA-RH7777 cells enhances VLDL assembly and secretion under lipid-rich conditions J Lipid Res 2010 51 150 161 10.1194/M900346-JLR200 19622837 PMC2789775

[B77] Tran K Thorne-Tjomsland G DeLong CJ Cui Z Shan J Burton L Jamieson JC Yao Z Intracellular assembly of very low density lipoproteins containing apolipoprotein B100 in rat hepatoma McA-RH7777 cells J Biol Chem 2002 277 31187 31200 10.1074/jbc.M200249200 12065576

[B78] Pan M Maitin V Parathath S Andreo U Lin SX St GC Yao Z Maxfield FR Williams KJ Fisher EA Presecretory oxidation, aggregation, and autophagic destruction of apoprotein-B: a pathway for late-stage quality control Proc Natl Acad Sci USA 2008 105 5862 5867 10.1073/pnas.0707460104 18391222 PMC2311371

[B79] Lopez-Soldado I Avella M Botham KM Differential influence of different dietary fatty acids on very low-density lipoprotein secretion when delivered to hepatocytes in chylomicron remnants Metabolism 2009 58 186 195 10.1016/j.metabol.2008.09.012 19154951 PMC2779336

[B80] Pal S Takechi R Ho SS Conjugated linoleic acid suppresses the secretion of atherogenic lipoproteins from human HepG2 liver cells Clin Chem Lab Med 2005 43 269 274 10.1515/CCLM.2005.045 15843229

[B81] Zhang P Boudyguina E Wilson MD Gebre AK Parks JS Echium oil reduces plasma lipids and hepatic lipogenic gene expression in apoB100-only LDL receptor knockout mice J Nutr Biochem 2008 19 655 663 10.1016/j.jnutbio.2007.08.005 18155507 PMC2610363

[B82] Werner A Havinga R Bos T Bloks VW Kuipers F Verkade HJ Essential fatty acid deficiency in mice is associated with hepatic steatosis and secretion of large VLDL particles Am J Physiol Gastrointest Liver Physiol 2005 288 G1150 G1158 10.1152/ajpgi.00456.2004 15662048

[B83] Degrace P Moindrot B Mohamed I Gresti J Du ZY Chardigny JM Sebedio JL Clouet P Upregulation of liver VLDL receptor and FAT/CD36 expression in LDLR-/- apoB100/100 mice fed trans-10,cis-12 conjugated linoleic acid J Lipid Res 2006 47 2647 2655 10.1194/jlr.M600140-JLR200 16957181

[B84] Ota T Gayet C Ginsberg HN Inhibition of apolipoprotein B100 secretion by lipid-induced hepatic endoplasmic reticulum stress in rodents J Clin Invest 2008 118 316 332 10.1172/JCI32752 18060040 PMC2104481

[B85] Su Q Tsai J Xu E Qiu W Bereczki E Santha M Adeli K Apolipoprotein B100 acts as a molecular link between lipid-induced endoplasmic reticulum stress and hepatic insulin resistance Hepatology 2009 50 77 84 10.1002/hep.22960 19434737

[B86] Carman GM Han GS Roles of phosphatidate phosphatase enzymes in lipid metabolism Trends Biochem Sci 2006 31 694 699 10.1016/j.tibs.2006.10.003 17079146 PMC1769311

[B87] Peterfy M Phan J Xu P Reue K Lipodystrophy in the fld mouse results from mutation of a new gene encoding a nuclear protein, lipin Nat Genet 2001 27 121 124 10.1038/83685 11138012

[B88] Donkor J Sariahmetoglu M Dewald J Brindley DN Reue K Three mammalian lipins act as phosphatidate phosphatases with distinct tissue expression patterns J Biol Chem 2007 282 3450 3457 10.1074/jbc.M610745200 17158099

[B89] Peterfy M Phan J Reue K Alternatively spliced lipin isoforms exhibit distinct expression pattern, subcellular localization, and role in adipogenesis J Biol Chem 2005 280 32883 32889 10.1074/jbc.M503885200 16049017

[B90] Manmontri B Sariahmetoglu M Donkor J Bou Khalil M Sundaram M Yao Z Reue K Lehner R Brindley DN Glucocorticoids and cyclic AMP selectively increase hepatic lipin-1 expression, and insulin acts antagonistically J Lipid Res 2008 49 1056 1067 10.1194/jlr.M800013-JLR200 18245816 PMC2311443

[B91] Bou Khlail M Sundaram M Zhang HY Links PH Raven JF Manmontri B Sariahmetoglu M Tran K Reue K Brindley DN The level and compartmentalization of phosphatidate phosphatase-1 (lipin-1) control the assembly and secretion of hepatic VLDL J Lipid Res 2009 50 47 58 18769019 10.1194/jlr.M800204-JLR200

[B92] Chen Z Gropler MC Norris J Lawrence JC Jr Harris TE Finck BN Alterations in hepatic metabolism in fld mice reveal a role for lipin 1 in regulating VLDL-triacylglyceride secretion Arterioscler Thromb Vasc Biol 2008 28 1738 1744 10.1161/ATVBAHA.108.171538 18669885 PMC2655237

[B93] Finck BN Gropler MC Chen Z Leone TC Croce MA Harris TE Lawrence JC Jr Kelly DP Lipin 1 is an inducible amplifier of the hepatic PGC-1alpha/PPARalpha regulatory pathway Cell Metab 2006 4 199 210 10.1016/j.cmet.2006.08.005 16950137

[B94] Cases S Stone SJ Zhou P Yen E Tow B Lardizabal KD Voelker T Farese RV Jr Cloning of DGAT2, a second mammalian diacylglycerol acyltransferase, and related family members J Biol Chem 2001 276 38870 38876 10.1074/jbc.M106219200 11481335

[B95] Cases S Smith SJ Zheng YW Myers HM Lear SR Sande E Novak S Collins C Welch CB Lusis AJ Identification of a gene encoding an acyl CoA:diacylglycerol acyltransferase, a key enzyme in triacylglycerol synthesis Proc Natl Acad Sci USA 1998 95 13018 13023 10.1073/pnas.95.22.13018 9789033 PMC23692

[B96] Smith SJ Cases S Jensen DR Chen HC Sande E Tow B Sanan DA Raber J Eckel RH Farese RV Jr Obesity resistance and multiple mechanisms of triglyceride synthesis in mice lacking Dgat Nat Genet 2000 25 87 90 10.1038/75651 10802663

[B97] Millar JS Stone SJ Tietge UJ Tow B Billheimer JT Wong JS Hamilton RL Farese RV Jr Rader DJ Short-term overexpression of DGAT1 or DGAT2 increases hepatic triglyceride but not VLDL triglyceride or apoB production J Lipid Res 2006 47 2297 2305 10.1194/jlr.M600213-JLR200 16877777

[B98] Liang JJ Oelkers P Guo C Chu PC Dixon JL Ginsberg HN Sturley SL Overexpression of human diacylglycerol acyltransferase 1, acyl-coa:cholesterol acyltransferase 1, or acyl-CoA:cholesterol acyltransferase 2 stimulates secretion of apolipoprotein B-containing lipoproteins in McA-RH7777 cells J Biol Chem 2004 279 44938 44944 10.1074/jbc.M408507200 15308631

[B99] Yamazaki T Sasaki E Kakinuma C Yano T Miura S Ezaki O Increased very low density lipoprotein secretion and gonadal fat mass in mice overexpressing liver DGAT1 J Biol Chem 2005 280 21506 21514 10.1074/jbc.M412989200 15797871

[B100] Liu Y Millar JS Cromley DA Graham M Crooke R Billheimer JT Rader DJ Knockdown of acyl-CoA:diacylglycerol acyltransferase 2 with antisense oligonucleotide reduces VLDL TG and ApoB secretion in mice Biochim Biophys Acta 2008 1781 97 104 18252207 10.1016/j.bbalip.2008.01.001

[B101] Walkey CJ Donohue LR Bronson R Agellon LB Vance DE Disruption of the murine gene encoding phosphatidylethanolamine N-methyltransferase Proc Natl Acad Sci USA 1997 94 12880 12885 10.1073/pnas.94.24.12880 9371769 PMC24232

[B102] Zhao Y Su B Jacobs RL Kennedy B Francis GA Waddington E Brosnan JT Vance JE Vance DE Lack of phosphatidylethanolamine N-methyltransferase alters plasma VLDL phospholipids and attenuates atherosclerosis in mice Arterioscler Thromb Vasc Biol 2009 29 1349 1355 10.1161/ATVBAHA.109.188672 19520976

[B103] Vance DE Vance JE Physiological consequences of disruption of mammalian phospholipid biosynthetic genes J Lipid Res 2009 50 Suppl S132 S137 10.1194/jlr.R800048-JLR200 18955728 PMC2674686

[B104] Jacobs RL Devlin C Tabas I Vance DE Targeted deletion of hepatic CTP:phosphocholine cytidylyltransferase alpha in mice decreases plasma high density and very low density lipoproteins J Biol Chem 2004 279 47402 47410 10.1074/jbc.M404027200 15331603

[B105] Jacobs RL Lingrell S Zhao Y Francis GA Vance DE Hepatic CTP:phosphocholine cytidylyltransferase-alpha is a critical predictor of plasma high density lipoprotein and very low density lipoprotein J Biol Chem 2008 283 2147 2155 10.1074/jbc.M706628200 18042552

[B106] Poloumienko A Cote A Quee AT Zhu L Bakovic M Genomic organization and differential splicing of the mouse and human Pcyt2 genes Gene 2004 325 145 155 10.1016/j.gene.2003.10.005 14697519

[B107] Fullerton MD Hakimuddin F Bonen A Bakovic M The development of a metabolic disease phenotype in CTP:phosphoethanolamine cytidylyltransferase-deficient mice J Biol Chem 2009 284 25704 25713 10.1074/jbc.M109.023846 19625253 PMC2757972

[B108] Li Z Agellon LB Allen TM Umeda M Jewell L Mason A Vance DE The ratio of phosphatidylcholine to phosphatidylethanolamine influences membrane integrity and steatohepatitis Cell Metab 2006 3 321 331 10.1016/j.cmet.2006.03.007 16679290

[B109] Gusarova V Brodsky JL Fisher EA Apolipoprotein B100 exit from the endoplasmic reticulum (ER) is COPII-dependent, and its lipidation to very low density lipoprotein occurs post-ER J Biol Chem 2003 278 48051 48058 10.1074/jbc.M306898200 12960170

[B110] Jones B Jones EL Bonney SA Patel HN Mensenkamp AR Eichenbaum-Voline S Rudling M Myrdal U Annesi G Naik S Mutations in a Sar1 GTPase of COPII vesicles are associated with lipid absorption disorders Nat Genet 2003 34 29 31 10.1038/ng1145 12692552

[B111] Asp L Magnusson B Rutberg M Li L Boren J Olofsson SO Role of ADP ribosylation factor 1 in the assembly and secretion of ApoB-100-containing lipoproteins Arterioscler Thromb Vasc Biol 2005 25 566 570 10.1161/01.ATV.0000154135.21689.47 15618550

[B112] Asp L Claesson C Boren J Olofsson SO ADP-ribosylation factor 1 and its activation of phospholipase D are important for the assembly of very low density lipoproteins J Biol Chem 2000 275 26285 26292 10.1074/jbc.M003520200 10843997

[B113] Rustaeus S Lindberg K Boren J Olofsson SO Brefeldin A reversibly inhibits the assembly of apoB containing lipoproteins in McA-RH7777 cells J Biol Chem 1995 270 28879 28886 10.1074/jbc.270.48.28879 7499415

[B114] Magnusson B Asp L Bostrom P Ruiz M Stillemark-Billton P Linden D Boren J Olofsson SO Adipocyte differentiation-related protein promotes fatty acid storage in cytosolic triglycerides and inhibits secretion of very low-density lipoproteins Arterioscler Thromb Vasc Biol 2006 26 1566 1571 10.1161/01.ATV.0000223345.11820.da 16627799

[B115] Ye J Li JZ Liu Y Li X Yang T Ma X Li Q Yao Z Li P Cideb, an ER- and lipid droplet-associated protein, mediates VLDL lipidation and maturation by interacting with apolipoprotein B Cell Metab 2009 9 177 190 10.1016/j.cmet.2008.12.013 19187774

[B116] Zhou Z Yon TS Chen Z Guo K Ng CP Ponniah S Lin SC Hong W Li P Cidea-deficient mice have lean phenotype and are resistant to obesity Nat Genet 2003 35 49 56 10.1038/ng1225 12910269

[B117] Kuipers F Jong MC Lin Y Eck M Havinga R Bloks V Verkade HJ Hofker MH Moshage H Berkel TJ Impaired secretion of very low density lipoprotein-triglycerides by apolipoprotein E- deficient mouse hepatocytes J Clin Invest 1997 100 2915 2922 10.1172/JCI119841 9389759 PMC508499

[B118] Mensenkamp AR Jong MC van GH Van Luyn MJ Bloks V Havinga R Voshol PJ Hofker MH van Dijk KW Havekes LM Apolipoprotein E participates in the regulation of very low density lipoprotein-triglyceride secretion by the liver J Biol Chem 1999 274 35711 35718 10.1074/jbc.274.50.35711 10585451

[B119] Maugeais C Tietge UJ Tsukamoto K Glick JM Rader DJ Hepatic apolipoprotein E expression promotes very low density lipoprotein-apolipoprotein B production in vivo in mice J Lipid Res 2000 41 1673 1679 11013310

[B120] Huang Y Liu XQ Rall SC Jr Taylor JM von EA Assmann G Mahley RW Overexpression and accumulation of apolipoprotein E as a cause of hypertriglyceridemia J Biol Chem 1998 273 26388 26393 10.1074/jbc.273.41.26388 9756870

[B121] Gusarova V Seo J Sullivan ML Watkins SC Brodsky JL Fisher EA Golgi-associated maturation of very low density lipoproteins involves conformational changes in apolipoprotein B, but is not dependent on apolipoprotein E J Biol Chem 2007 282 19453 19462 10.1074/jbc.M700475200 17500069

[B122] Gangabadage CS Zdunek J Tessari M Nilsson S Olivecrona G Wijmenga SS Structure and dynamics of human apolipoprotein CIII J Biol Chem 2008 283 17416 17427 10.1074/jbc.M800756200 18408013

[B123] Sundaram M Zhong S Bou Khalil M Zhou H Jiang ZG Zhao Y Iqbal J Hussain MM Figeys D Wang Y Functional analysis of the missense APOC3 mutation Ala23Thr associated with human hypotriglyceridemia J Lipid Res 2010 in press 10.1194/jlr.M005108 PMC3035516 20097930

[B124] Liu H Labeur C Xu CF Ferrell R Lins L Brasseur R Rosseneu M Weiss KM Humphries SE Talmud PJ Characterization of the lipid-binding properties and lipoprotein lipase inhibition of a novel apolipoprotein C-III variant Ala23Thr J Lipid Res 2000 41 1760 1771 11060345

[B125] Pennacchio LA Olivier M Hubacek JA Cohen JC Cox DR Fruchart JC Krauss RM Rubin EM An apolipoprotein influencing triglycerides in humans and mice revealed by comparative sequencing Science 2001 294 169 173 10.1126/science.1064852 11588264

[B126] van Dijk KW Rensen PC Voshol PJ Havekes LM The role and mode of action of apolipoproteins CIII and AV: synergistic actors in triglyceride metabolism? Curr Opin Lipidol 2004 15 239 246 10.1097/00041433-200406000-00002 15166778

[B127] Schaap FG Rensen PC Voshol PJ Vrins C van d V Chamuleau RA Havekes LM Groen AK van Dijk KW ApoAV reduces plasma triglycerides by inhibiting very low density lipoprotein-triglyceride (VLDL-TG) production and stimulating lipoprotein lipase-mediated VLDL-TG hydrolysis J Biol Chem 2004 279 27941 27947 10.1074/jbc.M403240200 15090553

[B128] Shu X Ryan RO Forte TM Intracellular lipid droplet targeting by apolipoprotein A-V requires the carboxyl-terminal segment J Lipid Res 2008 49 1670 1676 10.1194/jlr.M800111-JLR200 18450648 PMC2444012

[B129] Twisk J Gillian-Daniel DL Tebon A Wang L Barrett PH Attie AD The role of the LDL receptor in apolipoprotein B secretion J Clin Invest 2000 105 521 532 10.1172/JCI8623 10683382 PMC289165

[B130] James RW Martin B Pometta D Fruchart JC Duriez P Puchois P Farriaux JP Tacquet A Demant T Clegg RJ Apolipoprotein B metabolism in homozygous familial hypercholesterolemia J Lipid Res 1989 30 159 169 2715722

[B131] Teusink B Mensenkamp AR van der BH Kuipers F van Dijk KW Havekes LM Stimulation of the in vivo production of very low density lipoproteins by apolipoprotein E is independent of the presence of the low density lipoprotein receptor J Biol Chem 2001 276 40693 40697 10.1074/jbc.M106396200 11546779

[B132] Williams KJ Brocia RW Fisher EA The unstirred water layer as a site of control of apolipoprotein B secretion J Biol Chem 1990 265 16741 16744 2170353

[B133] Jiang XC Qin S Qiao C Kawano K Lin M Skold A Xiao X Tall AR Apolipoprotein B secretion and atherosclerosis are decreased in mice with phospholipid-transfer protein deficiency Nat Med 2001 7 847 852 10.1038/89977 11433351

[B134] Blasiole DA Oler AT Attie AD Regulation of ApoB secretion by the low density lipoprotein receptor requires exit from the endoplasmic reticulum and interaction with ApoE or ApoB J Biol Chem 2008 283 11374 11381 10.1074/jbc.M710457200 18272520 PMC2431081

[B135] Seidah NG Benjannet S Wickham L Marcinkiewicz J Jasmin SB Stifani S Basak A Prat A Chretien M The secretory proprotein convertase neural apoptosis-regulated convertase 1 (NARC-1): liver regeneration and neuronal differentiation Proc Natl Acad Sci USA 2003 100 928 933 10.1073/pnas.0335507100 12552133 PMC298703

[B136] Maxwell KN Breslow JL Adenoviral-mediated expression of Pcsk9 in mice results in a low-density lipoprotein receptor knockout phenotype Proc Natl Acad Sci USA 2004 101 7100 7105 10.1073/pnas.0402133101 15118091 PMC406472

[B137] Sun XM Eden ER Tosi I Neuwirth CK Wile D Naoumova RP Soutar AK Evidence for effect of mutant PCSK9 on apolipoprotein B secretion as the cause of unusually severe dominant hypercholesterolaemia Hum Mol Genet 2005 14 1161 1169 10.1093/hmg/ddi128 15772090

[B138] Lalanne F Lambert G Amar MJ Chetiveaux M Zair Y Jarnoux AL Ouguerram K Friburg J Seidah NG Brewer HB Jr Wild-type PCSK9 inhibits LDL clearance but does not affect apoB-containing lipoprotein production in mouse and cultured cells J Lipid Res 2005 46 1312 1319 10.1194/jlr.M400396-JLR200 15741654

[B139] Rashid S Curtis DE Garuti R Anderson NN Bashmakov Y Ho YK Hammer RE Moon YA Horton JD Decreased plasma cholesterol and hypersensitivity to statins in mice lacking Pcsk9 Proc Natl Acad Sci USA 2005 102 5374 5379 10.1073/pnas.0501652102 15805190 PMC556275

[B140] Clee SM Kastelein JJ van DM Marcil M Roomp K Zwarts KY Collins JA Roelants R Tamasawa N Stulc T Age and residual cholesterol efflux affect HDL cholesterol levels and coronary artery disease in ABCA1 heterozygotes J Clin Invest 2000 106 1263 1270 10.1172/JCI10727 11086027 PMC381437

[B141] Chung S Gebre AK Seo J Shelness GS Parks JS A novel role for ABCA1-generated large pre-beta migrating nascent HDL in the regulation of hepatic VLDL triglyceride secretion J Lipid Res 2010 51 729 742 10.1194/jlr.M900083 20215580 PMC2842152

[B142] Phung TL Roncone A Jensen KL Sparks CE Sparks JD Phosphoinositide 3-kinase activity is necessary for insulin-dependent inhibition of apolipoprotein B secretion by rat hepatocytes and localizes to the endoplasmic reticulum J Biol Chem 1997 272 30693 30702 10.1074/jbc.272.49.30693 9388205

[B143] Wiersma H Nijstad N Gautier T Iqbal J Kuipers F Hussain MM Tietge UJ Scavenger receptor BI facilitates hepatic very low density lipoprotein production in mice J Lipid Res 2010 51 544 553 10.1194/jlr.M000844 19723664 PMC2817584

[B144] Ginsberg HN New perspectives on atherogenesis: role of abnormal triglyceride-rich lipoprotein metabolism Circulation 2002 106 2137 2142 10.1161/01.CIR.0000035280.64322.31 12379586

[B145] Meshkani R Adeli K Hepatic insulin resistance, metabolic syndrome and cardiovascular disease Clin Biochem 2009 42 1331 1346 10.1016/j.clinbiochem.2009.05.018 19501581

[B146] Taghibiglou C Carpentier A Van Iderstine SC Chen B Rudy D Aiton A Lewis GF Adeli K Mechanisms of hepatic very low density lipoprotein overproduction in insulin resistance. Evidence for enhanced lipoprotein assembly, reduced intracellular ApoB degradation, and increased microsomal triglyceride transfer protein in a fructose-fed hamster model J Biol Chem 2000 275 8416 8425 10.1074/jbc.275.12.8416 10722675

[B147] Tsai J Zhang R Qiu W Su Q Naples M Adeli K Inflammatory NF-kappaB activation promotes hepatic apolipoprotein B100 secretion: evidence for a link between hepatic inflammation and lipoprotein production Am J Physiol Gastrointest Liver Physiol 2009 296 G1287 G1298 10.1152/ajpgi.90540.2008 19342510

[B148] Wiegman CH Bandsma RH Ouwens M Sluijs FH van der Havinga R Boer T Reijngoud DJ Romijn JA Kuipers F Hepatic VLDL production in ob/ob mice is not stimulated by massive de novo lipogenesis but is less sensitive to the suppressive effects of insulin Diabetes 2003 52 1081 1089 10.2337/diabetes.52.5.1081 12716736

[B149] Li X Grundy SM Patel SB Obesity in db and ob animals leads to impaired hepatic very low density lipoprotein secretion and differential secretion of apolipoprotein B-48 and B-100 J Lipid Res 1997 38 1277 1288 9254055

[B150] MacLean PS Bower JF Vadlamudi S Osborne JN Bradfield JF Burden HW Bensch WH Kauffman RF Barakat HA Cholesteryl ester transfer protein expression prevents diet-induced atherosclerotic lesions in male db/db mice Arterioscler Thromb Vasc Biol 2003 23 1412 1415 10.1161/01.ATV.0000080687.94313.67 12791674

[B151] Coenen KR Gruen ML Hasty AH Obesity causes very low density lipoprotein clearance defects in low-density lipoprotein receptor-deficient mice J Nutr Biochem 2007 18 727 735 10.1016/j.jnutbio.2006.12.010 17418556

[B152] Pickens MK Yan JS Ng RK Ogata H Grenert JP Beysen C Turner SM Maher JJ Dietary sucrose is essential to the development of liver injury in the MCD model of steatohepatitis J Lipid Res 2009 50 2072 2082 10.1194/jlr.M900022-JLR200 PMC2739762 19295183

[B153] Rinella ME Elias MS Smolak RR Fu T Borensztajn J Green RM Mechanisms of hepatic steatosis in mice fed a lipogenic methionine choline-deficient diet J Lipid Res 2008 49 1068 1076 10.1194/jlr.M800042-JLR200 18227531 PMC2311450

[B154] Yao ZM Vance DE The active synthesis of phosphatidylcholine is required for very low density lipoprotein secretion from rat hepatocytes J Biol Chem 1988 263 2998 3004 3343237

[B155] Pawar A Botolin D Mangelsdorf DJ Jump DB The role of liver × receptor-alpha in the fatty acid regulation of hepatic gene expression J Biol Chem 2003 278 40736 40743 10.1074/jbc.M307973200 12917410

[B156] Basciano H Miller A Baker C Naples M Adeli K LXRalpha activation perturbs hepatic insulin signaling and stimulates production of apolipoprotein B-containing lipoproteins Am J Physiol Gastrointest Liver Physiol 2009 297 G323 G332 10.1152/ajpgi.90546.2008 19497957

[B157] Huang W Dedousis N Bandi A Lopaschuk GD O'Doherty RM Liver triglyceride secretion and lipid oxidative metabolism are rapidly altered by leptin in vivo Endocrinology 2006 147 1480 1487 10.1210/en.2005-0731 16339207

[B158] Huang W Dedousis N O'Doherty RM Hepatic steatosis and plasma dyslipidemia induced by a high-sucrose diet are corrected by an acute leptin infusion J Appl Physiol 2007 102 2260 2265 10.1152/japplphysiol.01449.2006 17363621

[B159] Huang W Dedousis N Bhatt BA O'Doherty RM Impaired activation of phosphatidylinositol 3-kinase by leptin is a novel mechanism of hepatic leptin resistance in diet-induced obesity J Biol Chem 2004 279 21695 21700 10.1074/jbc.M401546200 14993225

[B160] Huang W Metlakunta A Dedousis N Ortmeyer HK Stefanovic-Racic M O'Doherty RM Leptin augments the acute suppressive effects of insulin on hepatic very low-density lipoprotein production in rats Endocrinology 2009 150 2169 2174 10.1210/en.2008-1271 19147673 PMC2671913

[B161] Chirieac DV Collins HL Cianci J Sparks JD Sparks CE Altered triglyceride-rich lipoprotein production in Zucker diabetic fatty rats Am J Physiol Endocrinol Metab 2004 287 E42 E49 10.1152/ajpendo.00297.2003 14970003

[B162] Taghibiglou C Carpentier A Van Iderstine SC Chen B Rudy D Aiton A Lewis GF Adeli K Mechanisms of hepatic very low density lipoprotein overproduction in insulin resistance. Evidence for enhanced lipoprotein assembly, reduced intracellular ApoB degradation, and increased microsomal triglyceride transfer protein in a fructose-fed hamster model J Biol Chem 2000 275 8416 8425 10.1074/jbc.275.12.8416 10722675

[B163] Benoist F Grand-Perret T Co-translational degradation of apolipoprotein B100 by the proteasome is prevented by microsomal triglyceride transfer protein. Synchronized translation studies on HepG2 cells treated with an inhibitor of microsomal triglyceride transfer protein J Biol Chem 1997 272 20435 20442 10.1074/jbc.272.33.20435 9252352

[B164] Fisher EA Zhou M Mitchell DM Wu X Omura S Wang H Goldberg AL Ginsberg HN The degradation of apolipoprotein B100 is mediated by the ubiquitin-proteasome pathway and involves heat shock protein 70 J Biol Chem 1997 272 20427 20434 10.1074/jbc.272.33.20427 9252351

[B165] Liao W Yeung SC Chan L Proteasome-mediated degradation of apolipoprotein B targets both nascent peptides cotranslationally before translocation and full-length apolipoprotein B after translocation into the endoplasmic reticulum J Biol Chem 1998 273 27225 27230 10.1074/jbc.273.42.27225 9765244

[B166] Ginsberg HN Role of lipid synthesis, chaperone proteins and proteasomes in the assembly and secretion of apoprotein B-containing lipoproteins from cultured liver cells Clin Exp Pharmacol Physiol 1997 24 A29 A32 10.1111/j.1440-1681.1997.tb03051.x 9143794

[B167] Ginsberg HN Fisher EA The ever-expanding role of degradation in the regulation of apolipoprotein B metabolism J Lipid Res 2009 50 Suppl S162 S166 10.1194/jlr.R800090-JLR200 19050312 PMC2674708

[B168] Shelness GS Ingram MF Huang XF DeLozier JA Apolipoprotein B in the rough endoplasmic reticulum: translation, translocation and the initiation of lipoprotein assembly J Nutr 1999 129 456S 462S 10064309 10.1093/jn/129.2.456S

[B169] Yao Z Tran K McLeod RS Intracellular degradation of newly synthesized apolipoprotein B J Lipid Res 1997 38 1937 1953 9374117

[B170] Mortimore GE Miotto G Venerando R Kadowaki M Autophagy Subcell Biochem 1996 27 93 135 8993159 10.1007/978-1-4615-5833-0_4

[B171] Seglen PO Berg TO Blankson H Fengsrud M Holen I Stromhaug PE Structural aspects of autophagy Adv Exp Med Biol 1996 389 103 111 8860999 10.1007/978-1-4613-0335-0_12

[B172] Cuervo AM Autophagy: many paths to the same end Mol Cell Biochem 2004 263 55 72 10.1023/B:MCBI.0000041848.57020.57 27520665

[B173] Ohsaki Y Cheng J Fujita A Tokumoto T Fujimoto T Cytoplasmic lipid droplets are sites of convergence of proteasomal and autophagic degradation of apolipoprotein B Mol Biol Cell 2006 17 2674 2683 10.1091/mbc.E05-07-0659 16597703 PMC1474802

